# Medulloblastoma uses GABA transaminase to survive in the cerebrospinal fluid microenvironment and promote leptomeningeal dissemination

**DOI:** 10.1016/j.celrep.2021.109302

**Published:** 2021-06-29

**Authors:** Vahan Martirosian, Krutika Deshpande, Hao Zhou, Keyue Shen, Kyle Smith, Paul Northcott, Michelle Lin, Vazgen Stepanosyan, Diganta Das, Jan Remsik, Danielle Isakov, Adrienne Boire, Henk De Feyter, Kyle Hurth, Shaobo Li, Joseph Wiemels, Brooke Nakamura, Ling Shao, Camelia Danilov, Thomas Chen, Josh Neman

**Affiliations:** 1 Department of Neurological Surgery, Keck School of Medicine, University of Southern California, Los Angeles, CA 90089, USA; 2 Department of Biomedical Engineering, Viterbi School of Engineering, University of Southern California, Los Angeles, CA 90089, USA; 3 Department of Developmental Neurobiology, St. Jude Children’s Research Hospital, Memphis, TN 38105, USA; 4 Human Oncology and Pathogenesis Program, Department of Neuro-Oncology, Memorial Sloan Kettering Cancer Center, New York, NY 10065, USA; 5 Magnetic Resonance Research Center, Department of Radiology and Biomedical Imaging, Yale University School of Medicine, New Haven, CT 06510, USA; 6 Department of Pathology, Keck School of Medicine, University of Southern California, Los Angeles, CA 90089, USA; 7 Center for Genetic Epidemiology, Department of Preventative Medicine, Keck School of Medicine, University of Southern California, Los Angeles, CA 90089, USA; 8 Division of Gastrointestinal and Liver Diseases, Department of Medicine, Keck School of Medicine, University of Southern California, Los Angeles, CA 90089, USA; 9 Norris Comprehensive Cancer Center, University of Southern California, Los Angeles, CA 90089, USA; 10 USC Brain Tumor Center, University of Southern California, Los Angeles, CA 90089, USA; 11 Lead contact

## Abstract

Medulloblastoma (MB) is a malignant pediatric brain tumor arising in the cerebellum. Although abnormal GABAergic receptor activation has been described in MB, studies have not yet elucidated the contribution of receptor-independent GABA metabolism to MB pathogenesis. We find primary MB tumors globally display decreased expression of GABA transaminase (ABAT), the protein responsible for GABA metabolism, compared with normal cerebellum. However, less aggressive WNT and SHH subtypes express higher *ABAT* levels compared with metastatic G3 and G4 tumors. We show that elevated ABAT expression results in increased GABA catabolism, decreased tumor cell proliferation, and induction of metabolic and histone characteristics mirroring GABAergic neurons. Our studies suggest ABAT expression fluctuates depending on metabolite changes in the tumor microenvironment, with nutrient-poor conditions upregulating ABAT expression. We find metastatic MB cells require ABAT to maintain viability in the metabolite-scarce cerebrospinal fluid by using GABA as an energy source substitute, thereby facilitating leptomeningeal metastasis formation.

## In brief

Through the lens of developmental neurobiology, Martirosian et al. interrogate the mechanisms promoting medulloblastoma metastasis. They identify a pathway correlated with metabolic and histone characteristics in both differentiated neural cells and medulloblastoma and show how this pathway can be exploited by disseminated medulloblastoma cells to form leptomeningeal metastases.

## Graphical Abstract

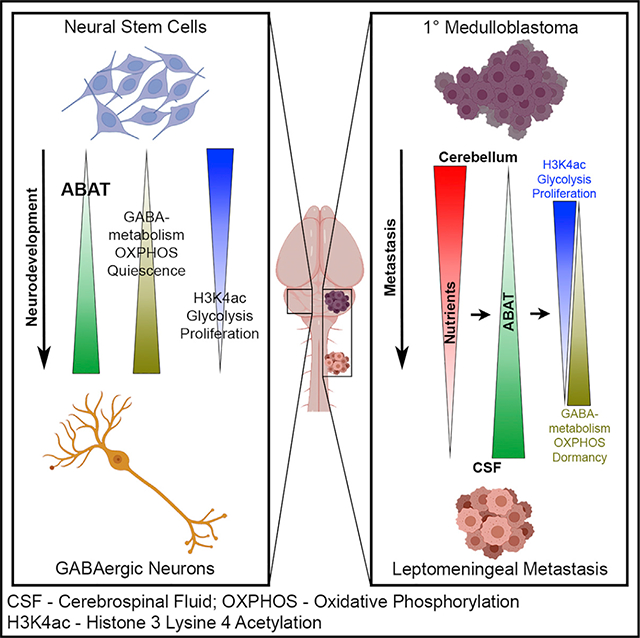

## INTRODUCTION

Central nervous system (CNS) malignancies are the most frequent form of solid tumors in pediatric patients (Ostrom et al., 2018) and remain the leading cause of cancer-related mortality in children (de Blank et al., 2015). Medulloblastoma (MB) is the most common of these neoplasms, presenting as an aggressive heterogeneous cerebellar lesion. MB is currently stratified into four genetically distinct subgroups: the wingless (WNT) group, the sonic hedgehog (SHH) group, group 3 (G3), and group 4 (G4) (Gilbertson and Ellison, 2008; Cavalli et al., 2017). Determining the biological mechanisms initiating oncogenesis has been elusive, because distinct somatically mutated driver genes in MB are rare (Jones et al., 2012; Northcott et al., 2012b). Consequently, the focus has shifted to analyzing MB formation through the scope of cerebellar development, with evidence showing that cerebellar development and MB tumorigenesis share a common genetic ancestry (Roussel and Hatten, 2011; Schüller et al., 2008; Yang et al., 2008). Recently, studies have elucidated how obstruction in neurodevelopmental programs gives rise to pediatric brain tumors (Jessa et al., 2019), and the transcriptional profile of these neoplasms mirrors those seen at specific time points of cerebellar development (Vladoiu et al., 2019). Therefore, cerebellar development can be used as a guide to discern the aberrant mechanisms in MB pathogenesis.

MB frequently forms intracranial metastases, specifically in patients diagnosed with G3 and G4 MBs (Liu et al., 2017; Northcott et al., 2012a). Treatment of patients with localized cerebellar tumors has been successful, with 78% of individuals achieving a cancer-free diagnosis (Zeltzer et al., 1999), albeit at the cost of significant long-term sequelae due to cytotoxic therapies (Sekeres et al., 2018; Mabbott et al., 2008). However, high-risk patients with leptomeningeal dissemination (LMD), including 40% of patients initially diagnosed with MB (Gajjar et al., 2006; Wu et al., 2012; Rutkowski et al., 2010), continue to have dismal outcomes (Fults et al., 2019), highlighting the need to study the mechanisms facilitating MB metastases. Metastasis research has grown to encompass the role of the metastatic tumor microenvironment (TME) in influencing malignant spread (Hanahan and Weinberg, 2011; Lambert et al., 2017). Although current MB LMD studies focus primarily on molecular changes in the primary tumor (Čančer et al., 2019; Grausam et al., 2017; Zhou et al., 2010; Kahn et al., 2018), there is scant evidence analyzing the influence of cerebrospinal fluid (CSF) on metastatic tumor characteristics in the leptomeningeal compartment.

γ-Aminobutyric acid (GABA), routinely described as an inhibitory neurotransmitter, is found in high concentrations in the neural parenchyma (Awapara et al., 1950; Roberts and Frankel, 1950) and has various roles in the CNS. As the cerebellum matures, quiescent neurons and astrocytes gain the ability to metabolize GABA as an energy source through the GABA shunt, a pathway mediated by the enzyme GABA transaminase (ABAT). ABAT has been studied mostly in plants and prokaryotes, whereas its ramifications in mammalian systems remain poorly understood. In the former, GABA synthesis and its catabolism through ABAT were found to be highly active during nutrient deprivation and environmental stresses (Feehily and Karatzas, 2013; Araújo et al., 2010), signifying its importance in cellular survival mechanisms. In mammals, reduced *ABAT* expression is implicated in primary clear cell renal carcinoma, breast cancer, and myelodysplastic syndrome tumorigenesis (Zhao et al., 2019; Lu et al., 2020; Chen et al., 2019). Furthermore, metastatic breast cancer cells in the neural parenchyma use GABA metabolism to form breast-to-brain metastases (Neman et al., 2014). In MB, genomic analyses have identified a neuronal signature for G3 and G4 subgroups, which express GABAergic and glutamatergic transcriptional profiles, respectively (Kool et al., 2008; Northcott et al., 2011; Cho et al., 2011). The GABAergic characterization in G3 tumors has been studied in regard to GABA receptors (Sengupta et al., 2014; Kallay et al., 2019), but GABA metabolism’s role and its implications in MB have not been elucidated.

Therefore, considering that MB formation shares mechanistic similarities with neurodevelopment, metastatic cells exploit GABA as an energy source in novel metabolic microenvironments, and disseminated cancer cells display a dormant phenotype (Wells et al., 2013; Giancotti, 2013) akin to neurons, we hypothesized that metastatic MB cells may exploit differentiated neural cells characteristics, such as GABA metabolism, to propagate LMD.

## RESULTS

### ABAT is localized in GABAergic neural cell mitochondria and is associated with a differentiated phenotype

**Figure 1.**
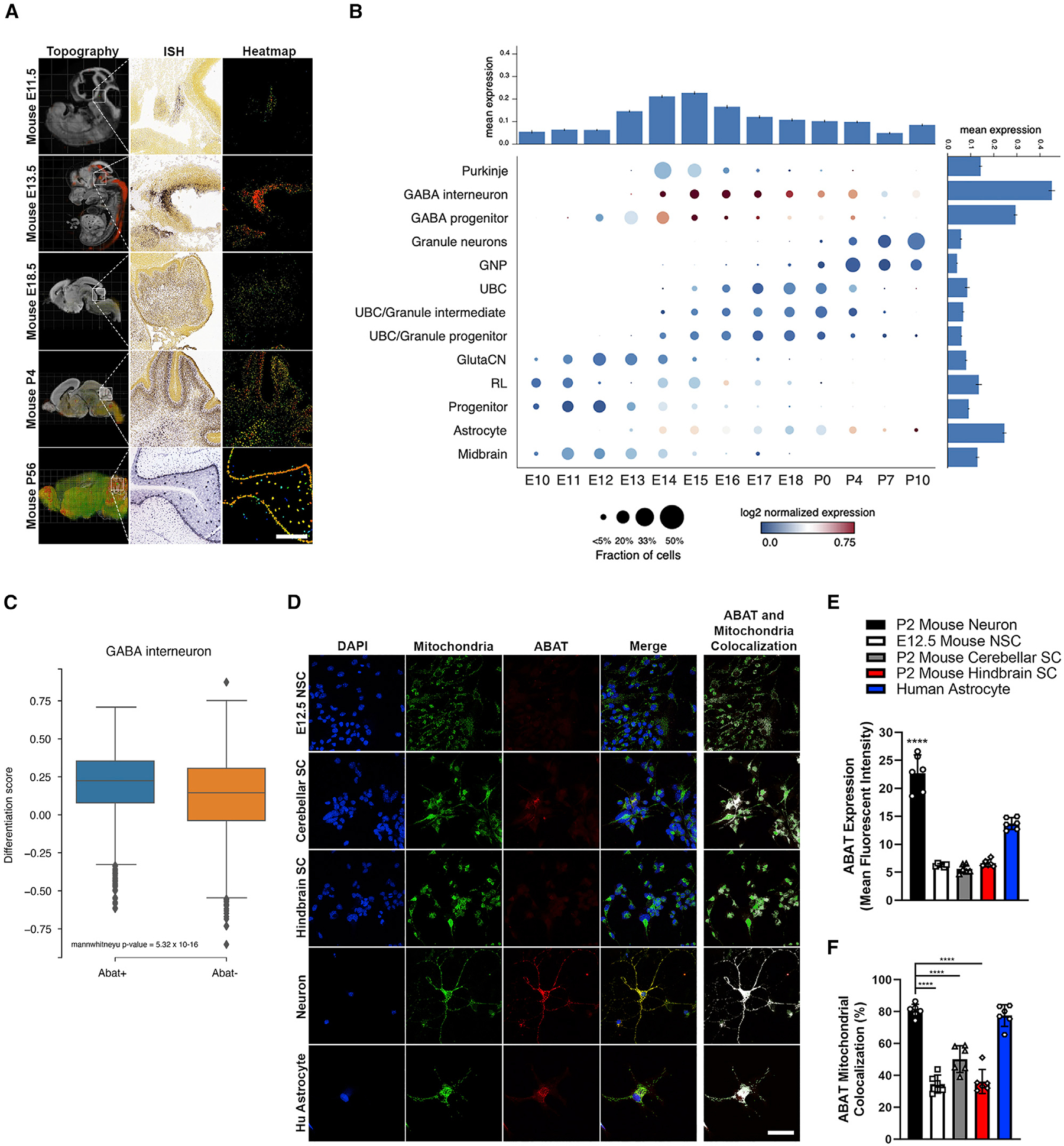
ABAT is localized in GABAergic neural cell mitochondria and is associated with a differentiated phenotype (A) *ABAT* topography and *in situ* hybridization (ISH) data from embryonic and post-natal stages reveal increased *ABAT* expression in the developed cerebellum. Scale bar, 250 μm. (B) Single-cell sequencing data from a developing mouse brain at different developmental stages show that *ABAT* expression is highest in GABA interneuron and progenitor populations. (C) *ABAT* expression within the GABA interneuron population is significantly associated with a higher differentiation score. p < 0.0001. Mann-Whitney U test. (D–F) Mouse neurons have the highest ABAT expression and mitochondrial localization among neural cells. Scale bar, 50 μm; one-way ANOVA followed by Tukey’s multiple comparison test, n = 6. NSC, neural stem cell; SC, stem cell. n = cells quantified. Histogram data depict mean ± SD. ****p < 0.0001. See also Figure S1.

To study how transdisciplinary investigation between neuroscience and cancer foundation can advance our understanding of MB, we sought to assess *ABAT* expression dynamics throughout neurodevelopment. Early neuroscience studies loosely linked ABAT with a differentiated state by showing higher ABAT activity in quiescent neurons compared with pre- and perinatal neural cells (Schousboe et al., 1989). *In situ* hybridization (ISH) data from the Allen Brain Atlas’s Developing Mouse Brain demonstrate that *ABAT* expression increases as neurodevelopment progresses (Figure 1A; Figure S1A). A sudden increase and subsequent decrease in *ABAT* is seen during the embryonic day (E) 11.5 to E13.5 developmental period, which coincides with the cerebellar development phase containing the highest proportion of reported GABAergic progenitors (Carter et al., 2018; Butts et al., 2014). To identify which neuron subtypes express *ABAT*, we used single-cell RNA (scRNA) sequencing data from the developing mouse brain (Carter et al., 2018). High *ABAT* expression is seen in the GABA interneuron and progenitor subtypes, with moderate expression in astrocytes (Figure 1B). In the GABA interneuron population, increased *ABAT* expression was significantly correlated with a higher differentiation score (Figure 1C). We then analyzed neural cell subtypes at various developmental stages *in vitro* to validate bioinformatic findings. Both *ABAT* and *ALDH5A1* (Figure S1B), the latter being the second protein in the GABA shunt cascade, were significantly higher in neurons compared with other cell types. Similarly, ABAT protein expression was significantly higher in neurons (Figures 1D and 1E). In contrast, ALDH5A1 protein expression was highest only in neurons compared to astrocytes (Figures S1C and S1D), indicating that ABAT expression is more correlated with neurodevelopment than is ALDH5A1. Because GABA is catabolized within the mitochondrial matrix (Schousboe et al., 1980), we next interrogated ABAT and ALDH5A1 mitochondrial localization in neural cell types. Results showed that mitochondrial ABAT is significantly higher in differentiated neurons and astrocytes compared with actively proliferating E12.5 neural stem cells and post-natal day (P) 2 cerebellar and hindbrain stem cells (Figures 1D and 1F). Although there was a significant decrease in ALDH5A1 mitochondrial localization in astrocytes compared with neurons, no significant difference was seen across other neural cell types (Figure S1E). Overall, these data suggest *ABAT* increases throughout neurodevelopment and has the highest expression in GABAergic cells. Moreover, cells that maintain a quiescent phenotype, like neurons, have higher ABAT expression and mitochondrial localization, whereas those that actively replicate, like stem cells, have reduced ABAT expression and mitochondrial localization.

### Higher *ABAT* expression is found in less aggressive MB subtypes and signifies reduced proliferative potential

**Figure 2.**
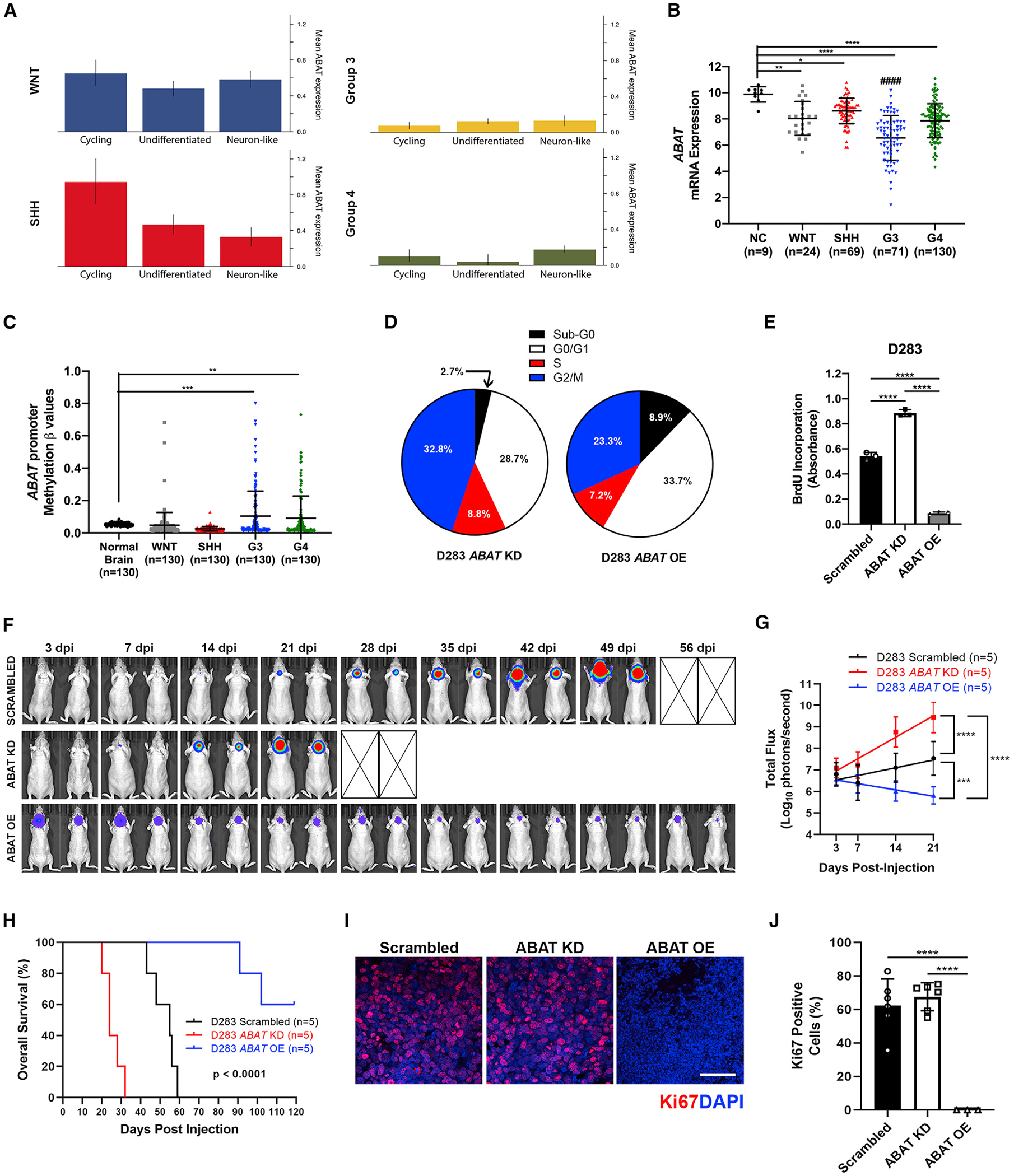
Higher *ABAT* expression is found in less aggressive MB subtypes and signifies reduced proliferative potential (A) Single-cell RNA sequencing analysis reveals *ABAT* does not characterize undifferentiated or neuron-like subtypes in primary MB tumors. (B) *ABAT* quantification in normal cerebella (NC) and MB (https://hgserver1.amc.nl/cgi-bin/r2/main.cgi) shows decreased expression in MB tumors. G3 tumors express the lowest *ABAT* levels among the MB subgroups, and less aggressive WNT and SHH have higher *ABAT* expression. One-way ANOVA. (C) Normal brain and MB tumor DNA methylation analysis in the *ABAT* promoter CpG islands shows significant promoter methylation increase in G3 and G4 MBs. One-way ANOVA. (D) Cell-cycle analysis shows a higher percentage of ABAT OE cells in the sub-G0 and G0/G1 phases, whereas ABAT KD cells are in the S and G2/M cell-cycle stages. (E) BrdU incorporation assay shows ABAT OE cells proliferate significantly more slowly than ABAT KD cells. One-way ANOVA, n = 6. (F) Bioluminescent imaging (BLI) indicates that ABAT OE tumors are not viable in the cerebellum, whereas ABAT KD cells grow rapidly. BLI for the ABAT OE group (n = 5) was analyzed at an increased sensitivity, because imaging at the Scrambled/ABAT KD group (n = 5) sensitivity scale would not show the presence and subsequent decrease of tumor burden in the ABAT OE group. (G) BLI total flux quantification reveals that ABAT OE tumors grow significantly more slowly. Two-way ANOVA. (H) Kaplan-Meier curves show ABAT OE xenografts have increased survival. p < 0.0001. Log-rank (Mantel-Cox) test. (I and J) Scrambled and ABAT KD tumors (n = 6) show significantly higher Ki67-positive cells compared with ABAT OE tumors (n = 3). Scale bar, 50 μm; one-way ANOVA. n = replicates (E), animals per group (F), or regions of interest analyzed (J). Histogram data depict mean ± SD. *p < 0.05, **p < 0.01, ***p < 0.001, ****p < 0.0001, ####p < 0.0001. One- and two-way ANOVAs followed by Tukey’s multiple comparison test (B, C, G, and J). See also Figure S2.

Recent studies have elucidated varying differentiation levels across cells in MB tumors (Vladoiu et al., 2019). Thus, we sought to determine whether *ABAT* expression in primary MB denotes a specific differentiation state. To query this, we analyzed the primary MB scRNA sequencing dataset (Hovestadt et al., 2019). Results showed that *ABAT* expression does not affect primary MB differentiation status, because *ABAT*-expressing cells can exhibit both undifferentiated and neuron-like states, regardless of molecular subgroup (Figure 2A). We next interrogated *ABAT* expression among MB subgroups relative to normal brain tissue. Although expression of ABAT (both mRNA and protein) is significantly decreased in all MB molecular and histological subgroups compared with normal tissue (Figure 2B; Figures S2A and S2B), A*BAT* expression is higher in the less aggressive WNT and SHH subtypes compared with the more aggressive G3 and G4 subtypes (Figures 2A and 2B). Using whole-genome sequencing datasets (Cerami et al., 2012; Gao et al., 2013), we determined *ABAT* expression decrease is not caused by a mutation at the *ABAT* locus (Figure S2C). Subsequently, we considered epigenetic regulation in *ABAT* transcript reduction. We analyzed methylation data from 1,256 patients with MB (Northcott et al., 2017) and elucidated a significantly higher *ABAT* promoter methylation in G3 and G4 MBs compared with WNT MB, SHH MB, and normal brain tissues (Figure 2C). This finding provides evidence that epigenetic mechanisms in G3 and G4 MBs contribute to decreased *ABAT* expression in these subgroups.

Considering that *ABAT* expression was higher in the less aggressive MB subtypes, we next established stable lentiviral knockdown (KD) and overexpression (OE) models (Figures S2D and S2E) to determine whether *ABAT* expression is implicated in cell proliferation capabilities. Five *ABAT* KD variants were prepared and analyzed, and the 927 variant was chosen for further experiments (Figure S2D). We first performed cell-cycle analysis to determine whether modulating *ABAT* alters cell-cycle progression. Results showed that ABAT OE cells are predominantly in the sub-G0 and G0/G1 phases, whereas ABAT KD cells have a higher percentage of cells in the S and G2/M phases (Figure 2D). ABAT OE cells also express a significant reduction in cell-cycle mediators *CCNB2*, *CCNF*, *CDC25A*, *CDC25C*, and *CDK1* yet a significant increase in cell-cycle inhibitors *CDKN1A* and *CDKN2A* (Figures S2F and S2G). We then performed bromodeoxyuridine (BrdU) incorporation assays to provide further evidence that *ABAT* modulation can mediate cell proliferation. Our data demonstrated that D283 and D425 ABAT OE cells had a significant decrease in growth rate compared with ABAT KD cells (Figure 2E; Figure S2H). To verify these findings *in vivo*, we transplanted 200,000 D283 Scrambled, ABAT KD, and ABAT OE MB cells into the cerebella of mice. Bioluminescent imaging (BLI) revealed that ABAT OE tumors display the slowest growth rate in the cerebellum (Figures 2F and 2G). Moreover, animals in the ABAT OE group display a significant increase in overall survival compared with the scrambled and ABAT KD groups (Figure 2H). Kaplan-Meier curves from patients with MB displayed a similar pattern (Figure S2I). To quantify these differences in growth, we stained MB tissues with proliferation marker Ki67. Analysis showed significant Ki67 reduction in the ABAT OE group, providing evidence that MB cells harboring increased *ABAT* have reduced proliferation in the cerebellar microenvironment (Figures 2I and 2J). Altogether, these data suggest primary MB tumors have reduced *ABAT* expression compared with normal cerebellum, and less aggressive MB subtypes (WNT and SHH) express significantly higher *ABAT* expression compared with their more aggressive subtypes (G3). Furthermore, increased *ABAT* expression induces inhibitory effects on the cell cycle, reduces cell proliferation, and signifies greater overall survival for mice with cerebellar MB tumors.

### MB tumors express heterogenous ABAT, which increases in nutrient-scarce microenvironments

**Figure 3.**
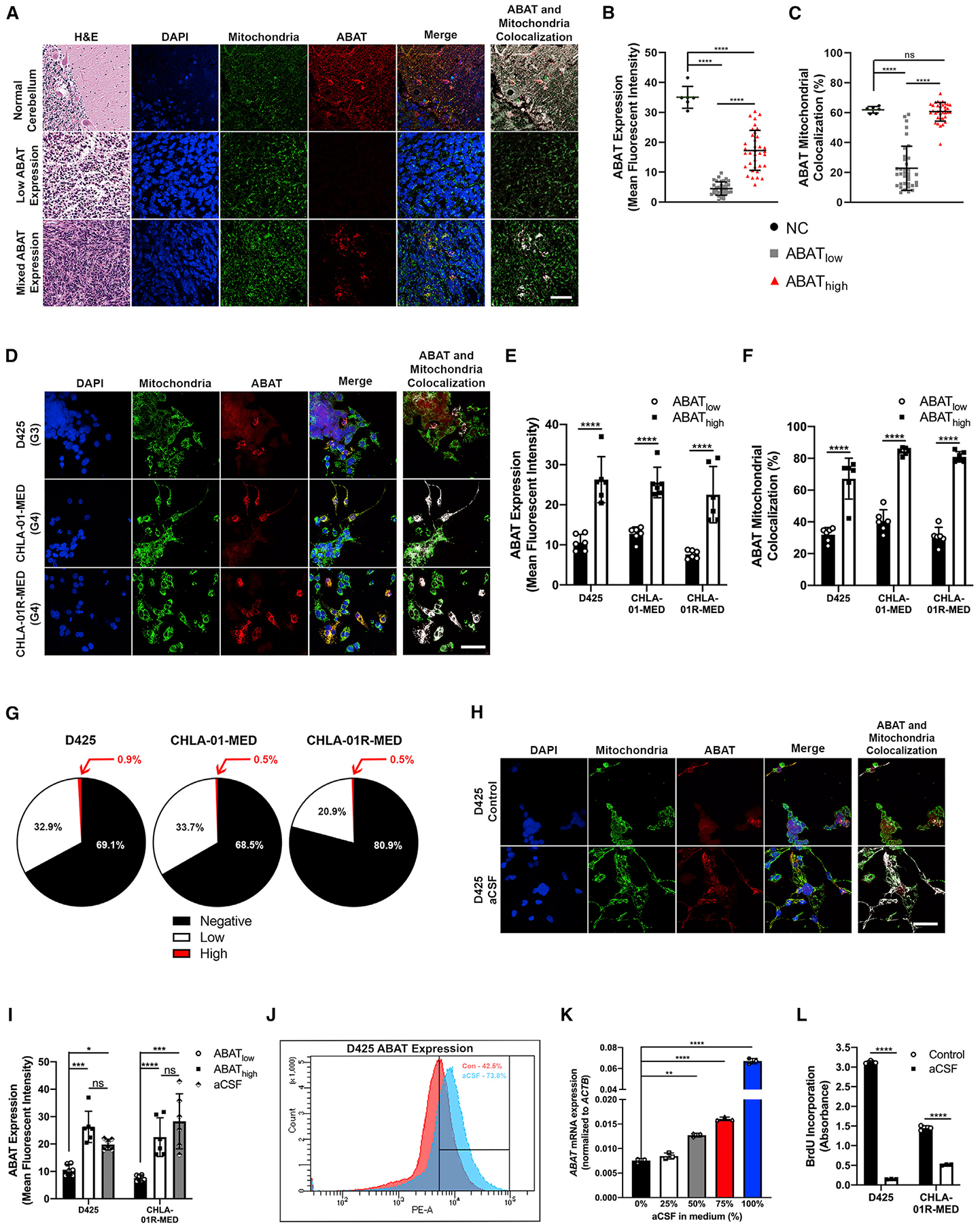
MB tumors express heterogenous ABAT, which increases in nutrient-scarce microenvironments (A) Representative ABAT immunofluorescence staining reveals that 6/18 tumors exhibit mixed ABAT expression. Scale bar, 50 μm. (B and C) ABAT expression and mitochondrial localization analysis of NC (n = 6) and ABAT_high_/ABAT_low_ regions of interest (n = 36) in MB tumors show significantly higher ABAT expression and mitochondrial localization in NC and ABAT_high_ MB. One-way ANOVA. (D) D425, CHLA-01-MED, and CHLA-01R-MED cells exhibit heterogeneous ABAT expression. Scale bar, 50 μm. (E and F) ABAT_high_ cells display a significant increase in ABAT expression and mitochondrial localization. t test, n = 6. (G) Flow cytometry analysis reveals that ABAT_high_ cells are a rare subset of cells in the tumor bulk. (H) D425 cells in artificial CSF (aCSF) medium display increased ABAT expression. Scale bar, 50 μm. (I) aCSF cells display similar ABAT levels compared with ABAT_high_ cells in control medium. Two-way ANOVA, n = 6. (J) ABAT flow cytometry analysis reveals that aCSF cells have higher overall ABAT expression. (K) Increasing aCSF concentration in medium results in a stepwise increase in *ABAT* expression. One-way ANOVA. (L) BrdU incorporation analysis reveals that aCSF cells have a significant decrease in proliferation rate. t test, n = 3. n = cells/regions of interest (ROIs) quantified (B, C, F, and I) or replicates (L). Histogram data depict mean ± SD. *p < 0.05, **p < 0.01, ***p < 0.001, ****p < 0.0001. One- and two-way ANOVAs followed by Tukey’s multiple comparison test (B, C, I, and K). See also Figure S3.

MB is a highly heterogeneous tumor; thus, we investigated whether ABAT is also heterogeneously expressed. We first queried ABAT expression in human cerebellar and MB tissues. As expected, normal cerebellum expressed high ABAT levels localized in the mitochondria (Figure 3A). Conversely, although most MB samples were negative for ABAT, we observed rare ABAT-expressing cells and stratified all MB histological tissue by either low-ABAT or mixed-ABAT expression (Figure 3A). Patient tissues with mixed-ABAT expression were further categorized as having ABAT_low_ or ABAT_high_ cells. Overall, ABAT expression is significantly higher in the normal cerebellum compared with both ABAT_low_ and ABAT_high_ cells in MB tissue; however, ABAT_high_ cells have significantly higher ABAT compared with ABAT_low_ cells (Figure 3B). Normal cerebellar and ABAT_high_ cells showed no significant difference in ABAT mitochondrial localization, and both had significantly higher mitochondrial localization than ABAT_low_ cells (Figure 3C). *In vitro*, ABAT-expressing cells were found in the D425, CHLA-01-MED, and CHLA-01R-MED cell lines but were absent from the UW228-2 and D283 lines (Figure 3D; Figure S3A). Furthermore, ABAT_high_ cells demonstrate significantly higher ABAT expression and mitochondrial localization compared with ABAT_low_ cells (Figures 3E and 3F). Orthotopic transplantation of D283 and Med-2112FH MB cells into mice cerebella recapitulated mixed ABAT expression seen in patient tissues and *in vitro* (Figure S3B). Conversely, ALDH5A1 was expressed ubiquitously across all cells (Figure S3C). To determine the frequency of ABAT_high_-expressing cells, we performed fluorescence-activated cell sorting (FACS) analysis on D425, CHLA-01-MED, and CHLA-01R-MED cell lines. Consistent with our findings in patient tissues, 68.5%–80.9% of cells expressed ABAT at background levels and 20.9%–32.9% expressed low levels of the protein. Moreover, ABAT_high_ cells represented a rare subpopulation of the tumor, with only 0.5%–0.9% of cells expressing high levels (Figure 3G). Considering that ABAT functionality increases in nutrient-depleted environments (Feehily and Karatzas, 2013; Araújo et al., 2010), we hypothesized that ABAT_high_ cells would be better adapted to survive in such conditions. We speculated that metastatic MB cells encounter such an environment in the CSF, where they would possibly increase ABAT expression to survive in the metabolite-scare CSF and facilitate metastasis formation. We first analyzed *SOX9* expression, because rare SOX9-positive cells propagate tumor recurrence and metastasis in MB (Swartling et al., 2014). Results showed a significant increase in *SOX9* expression in ABAT OE cells (Figure S3D). We next considered analyzing LMD samples from patients with MB to query *ABAT* expression. Because LMD samples from pediatric patient CSF were not readily available, we analyzed *ABAT* and *ALDH5A1* expression in LMD samples from the CSF of patients with breast and lung cancer as a surrogate. Results showed that LMDs have high *ABAT* and *ALDH5A1* expression (Figure S3E), indicating that ABAT may serve a vital role for metastatic tumor cells in the CSF. Thus, we established an artificial CSF (aCSF) condition, using metabolite concentrations derived from MB patient CSF (Benoist et al., 2003; el-Yazigi and Al-Mefty, 1985; Goldsmith et al., 1987), to elucidate whether MB cells upregulate ABAT in the leptomeningeal microenvironment *in vitro*. D425 and CHLA-01R-MED MB cells in aCSF displayed significant *ABAT* upregulation, whereas only D425 cells had elevated *ALDH5A1 in vitro* (Figure S3F). Similarly, ABAT expression significantly increased in aCSF (Figures 3H and 3I; Figure S3G), and ABAT mitochondrial localization mirrored ABAT_high_ cells (Figure S3H). Furthermore, we analyzed ABAT expression in control and aCSF using flow cytometry analysis. Results showed an increase in ABAT-expressing cells in aCSF condition (Figure 3J; Figure S3I). Titration of aCSF medium showed a stepwise *ABAT* increase, indicating that changes in nutrient availability in the TME influence ABAT levels (Figure 3K). Subsequently, we observed increased GABA in the mitochondria of aCSF cells (Figure S3J), suggesting that GABA is shuttled to this subcellular compartment for metabolic use. We next analyzed proliferation changes in aCSF-conditioned cells to determine whether increased ABAT expression induced a decrease in growth rate. BrdU incorporation analysis and Ki67 staining revealed a significant decrease in proliferative capabilities in MB cells under aCSF conditions (Figure 3L; Figures S3K and S3L). The observed differences in growth rate prompted us to determine whether ABAT-expression gain results in chemo-resistance to cisplatin and vincristine (Bates and Eastman, 2017; Basu and Krishnamurthy, 2010), which are common therapies used in patients with MB (Martin et al., 2014). We first established half maximal inhibitory concentration (IC_50_) values for both drugs in control conditions (Figures S3M–S3O). We next tested ABAT KD and OE cell viability treated at IC_50_. In both D283 and D425 cells, ABAT OE cells were more resistant to vincristine treatment than ABAT KD cells *in vitro*. However, although D425 ABAT OE cells displayed similar resistance to cisplatin, D283 ABAT KD cells had higher resistance to cisplatin than their ABAT OE counterparts (Figure S3P). Likewise, D425 and CHLA-01R-MED cells cultured in aCSF have significantly higher resistance than controls to both drugs (Figure S3Q). Altogether, we provide evidence that MB tumors display heterogenous ABAT expression that increases in nutrient-scarce microenvironments and induces a decrease in proliferation.

### MB cells require ABAT to form leptomeningeal metastases

**Figure 4.**
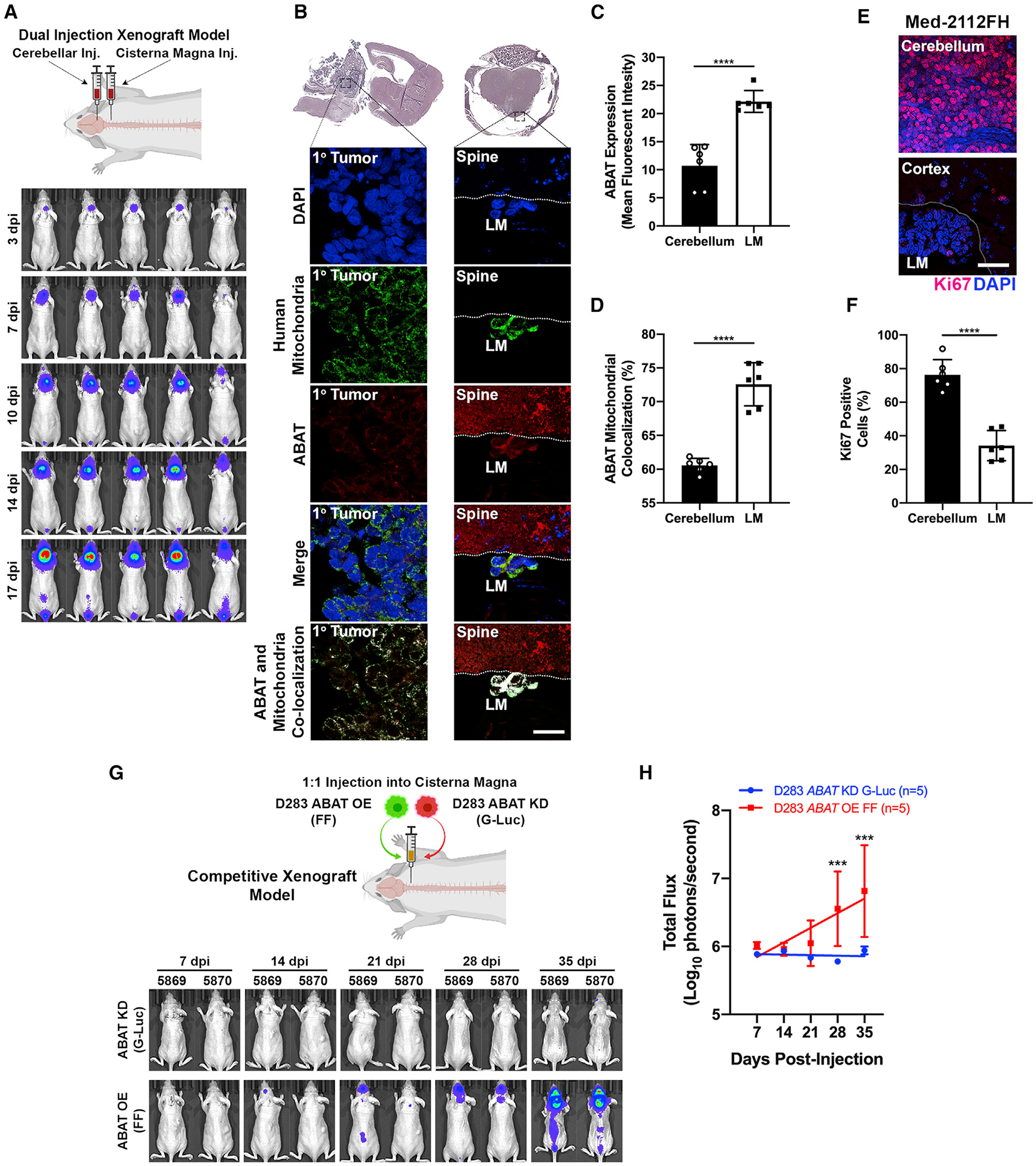
MB cells require ABAT to form leptomeningeal metastases (A) BLI of mice with cerebellar and cisterna magna injections shows primary and metastatic tumors. n = 5. (B–D) Leptomeningeal metastases (LMs) show increased ABAT expression and mitochondrial localization compared with cells in the cerebellum. Scale bar, 20 μm; t test, n = 6. (E and F) LMs show decreased Ki67 positivity. Scale bar, 50 μm; t test, n = 6. (G and H) Equal ratio of ABAT KD and ABAT OE cells transplanted into the cisterna magna of a single mouse. ABAT OE cells form LM, whereas their ABAT KD counterparts cannot. Two-way ANOVA followed by Tukey’s multiple comparison test. n = xenografted animals (A), cells (C and D), or images quantified (F) or animals per group (G and H). Histogram data depict mean ± SD. *p < 0.05, **p < 0.01, ***p < 0.001, ****p < 0.0001. Xenograft illustrations created with BioRender.com.

To determine whether ABAT expression is required by MB cells to survive in the nutrient-deficient CSF microenvironment, we established a clinically relevant xenograft model to recapitulate a patient who presents with both cerebellar mass and LMD. Mice were xenografted with 100,000 MB cells in both the cerebellum and the cisterna magna. Tumor-bearing mice presented with distinct cerebellar tumors, as well as LMD in the lumbar and sacral region (Figure 4A). We then analyzed ABAT expression in the cerebellar and metastatic tumor sites 21 days post-tumor injection, thereby interrogating the initial stages of metastasis formation. Results showed that ABAT is significantly upregulated in LMD samples relative to the primary cerebellar tumor (Figures 4B and 4C). Furthermore, metastases express higher mitochondrial ABAT (Figures 4B and 4D). Metastatic cells are frequently associated with a dormant state (Wells et al., 2013; Giancotti, 2013). Thus, we analyzed Ki67 expression in both the cerebellar and the leptomeningeal compartments. MB cells in the leptomeningeal compartment displayed significantly reduced Ki67 positivity compared with MB cells in the cerebellum (Figures 4E and 4F). To elucidate whether ABAT expression is necessary for MB cells to seed the leptomeningeal space, we established a competitive xenograft model in which we transplanted 200,000 ABAT KD cells (under the Gaussia luciferase [G-luc] reporter) and ABAT OE cells (under the Firefly [FF] luciferase reporter) into the cisterna magna at a 1:1 ratio (100,000 cells each) into the same animal. Results showed that ABAT KD cells were unable to seed the CSF and form LMD, whereas ABAT OE cells were able to form leptomeningeal metastases in the CNS (Figures 4G and 4H). Thus, we provide evidence that ABAT is critical for MB cells to form LMD.

### Higher ABAT expression increases GABA metabolism, promotes survival in nutrient-poor conditions, and induces an oxidative phosphorylation (OXPHOS OXPHOS) metabolic phenotype

**Figure 5.**
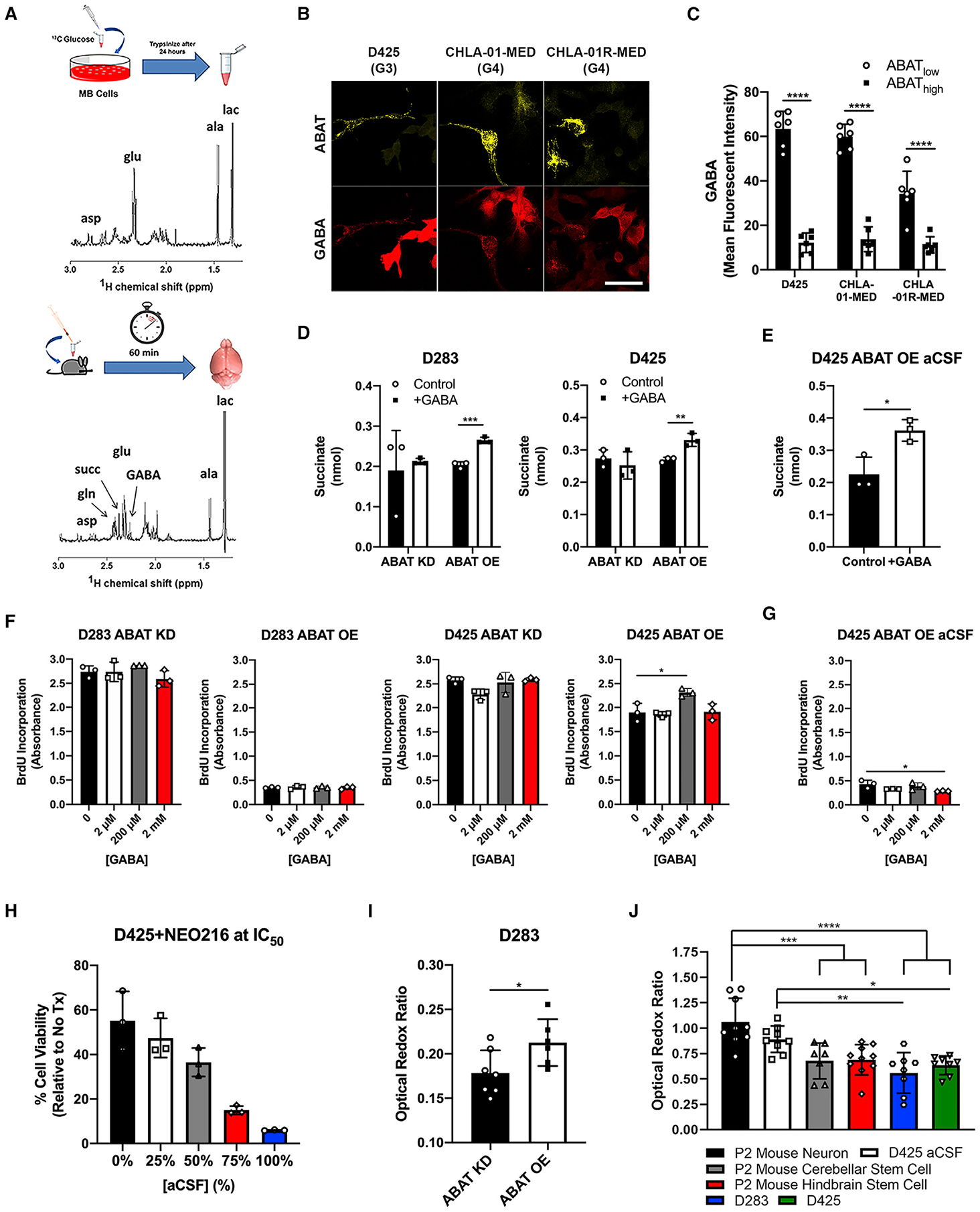
Higher ABAT expression increases GABA metabolism, promotes survival in nutrient-poor conditions, and induces an OXPHOS metabolic phenotype (A) Magnetic resonance spectroscopy analysis indicates that GABA and the GABA shunt metabolites glutamate and succinate are present in the MB tumor microenvironment. (B and C) Cells given exogenous GABA show accumulation of GABA in ABAT_low_ cells. Scale bar, 20 μm; t test, n = 6. (D) D283 and D425 ABAT OE cells show an increase in the GABA catabolism product succinate after exogenous supraphysiological GABA treatment, whereas ABAT KD cells show no significant change. t test, n = 3. (E) ABAT OE cells in aCSF show a significant increase in succinate. t test, n = 3. (F and G) Exogenous treatment of supraphysiological GABA levels shows no significant change in proliferation rate, excluding significance at 200 μM in D425 ABAT OE cells and at 2 mM in D425 ABAT OE aCSF cells. One-way ANOVA, n = 3. (H) Increasing aCSF concentration leads to increased MB sensitivity to ABAT inhibitor NEO216. n = 3. (I) Optical redox ratio analysis using a two-photon confocal microscope shows ABAT OE cells (n = 6) have higher OXPHOS utilization than ABAT KD cells (n = 7). t test. (J) D425 aCSF cells (n = 9) display a optical redox ratio similar to that of neurons (n = 9), a higher ratio compared with cerebellar (n = 7) and hindbrain (n = 10) stem cells, and a significantly higher ratio compared with D283 (n = 8) and D425 (n = 8) cells in control medium. One-way ANOVA. n = number of cells quantified (C), replicates (D–H), or images analyzed (I and J). Histogram data depict mean ± SD. *p < 0.05, **p < 0.01, ***p < 0.001, ****p < 0.0001. One-way ANOVA followed by Tukey’s multiple comparison test (F, G, and J). See also Figure S4.

We next determined the mechanism behind ABAT promoting LMD in MB. We hypothesized that the TME has an important role in regulating GABA metabolism. We first treated *in vitro* MB samples and MB xenografts (14 days post-injection) with C-13-labeled glucose and analyzed GABA levels using magnetic resonance spectroscopy. *In vivo*, endemic to their initiation site, GABA and GABA shunt metabolites glutamate and succinate were readily detected within tumors, whereas *in vitro* analysis of cells determined that GABA was absent (Figure 5A), indicating the importance of the TME in mediating GABA production and metabolism. Next, we treated *in vitro* MB cells with GABA and analyzed the effects on GABA shunt levels. *ABAT* expression was not altered across MB cells, whereas D425 cells showed a four-fold increase in *ALDH5A1* expression (Figure S4A). Next, we determined whether GABA catabolism was done specifically by ABAT_high_ cells. We treated tumor cells expressing both ABAT_high_ and ABAT_low_ subpopulations with supraphysiological GABA concentrations and then quantified the neurotransmitter’s levels in each cell type. Results showed that ABAT_low_ cells have significantly higher GABA levels compared with ABAT_high_ cells (Figures 5B and 5C). To provide evidence that ABAT_high_ cells metabolize GABA, we treated control, ABAT KD, and ABAT OE cells with GABA and analyzed intracellular succinate, a product of GABA catabolism. Control cells were unable to produce significantly increased succinate levels, an expected result considering that ABAT_high_ cells are a rare subpopulation among the tumor bulk (Figure S4B). Conversely, ABAT OE cells showed a significant succinate increase post-GABA treatment (Figure 5D). We next analyzed succinate in MB cells cultured in aCSF. Although control D425 cells did not exhibit significant succinate increase post-GABA treatment (Figure S4C), we did observe a significant increase in mitochondrial NADH, another product of GABA metabolism (Figures S4D–S4F), using a two-photon confocal microscope targeting endogenous fluorophores (i.e., NADH and FAD). We quantified mitochondrial NADH by using FAD as a surrogate marker for mitochondria (Heikal, 2010). However, D425 ABAT OE cells cultured in aCSF showed a significant increase in succinate after GABA treatment (Figure 5E), underscoring ABAT’s importance in facilitating GABA metabolism. Considering that metabolism overall is linked with changes in proliferation, we asked whether GABA treatment would alter cellular growth rate. Control cells treated with increasing GABA concentrations showed no significant changes in BrdU incorporation (Figure S3G). Similarly, ABAT KD and ABAT OE cell lines did not show growth changes, excluding a growth increase in the D425 ABAT OE cell line at 200 μM GABA (Figure 5F). Moreover, D425 ABAT OE cells cultured in aCSF were treated with GABA, and again increase in proliferation was not found (Figure 5G). Overall, these results indicate that GABA metabolism does not alter MB tumor cell proliferation in both high- and low-nutrient microenvironments. Therefore, we asked whether ABAT and GABA metabolism are important in promoting survival, rather than proliferation in nutrient-depleted conditions. To test this, we used a valproic acid derivative, NEO216 (Figure S4H). Compared with its parent molecule, which is known to inhibit histone deacetylase (HDAC) activity (Phiel et al., 2001), NEO216 displays reduced HDAC inhibition properties (Figure S4I). However, NEO216 showed direct ABAT inhibition, because cells with higher *ABAT* expression were more sensitive to this agent (Figure S4J). Furthermore, D283 ABAT KD and OE cells were treated with NEO216 at IC_50_ derived from control cells (Figure S4K). ABAT OE cells were significantly more sensitive to NEO216 compared with ABAT KD counterparts (Figure S4L). Thus, to determine the importance of ABAT in promoting survival in the CSF microenvironment and whether NEO216 could target these cells, we treated D425 cells cultured in increasing aCSF concentrations with NEO216 at IC_50_ (Figure S4M). *ABAT* induction with increasing aCSF concentrations (Figure 3K) resulted in greater NEO216 sensitivity in MB cells (Figure 5H).

Moreover, cells under stressful environments, including disseminated cancer cells (Weber, 2016; Pascual et al., 2018), revert to OXPHOS (Duan et al., 2018; Wu et al., 2016) to maintain necessary ATP levels and survive. Therefore, we evaluated the redox state in ABAT KD and OE cells. Using a two-photon confocal microscope, we analyzed the optical redox ratio (fluorescent intensity ratio between FAD and NADH), which at higher levels is indicative of an OXPHOS-dependent metabolic phenotype (Hou et al., 2016; Zhou et al., 2020). Results showed that ABAT OE cells have a significantly higher optical redox ratio compared with ABAT KD cells (Figure 5I). Furthermore, we queried the metabolic state in control and aCSF-cultured MB cells. Mouse neurons and stem cells were used as controls for an OXPHOS (Lange et al., 2016; Beckervordersandforth et al., 2017) and a glycolytic (Ito and Suda, 2014; Gu et al., 2016) metabolic condition, respectively. As expected, results showed that neurons express significantly higher levels of OXPHOS compared with stem cells (Figure 5J). Furthermore, MB cells in control medium did not have a significantly different metabolic phenotype compared with stem cells, which was expected considering that cancer cells are heavily reliant on a glycolysis-driven energy production cascade (Yu et al., 2017). Importantly, MB cells in aCSF medium shared similar OXPHOS levels with neurons and had significantly higher levels compared with control MB cells. We then considered whether MB cells in the leptomeningeal space acquire paracrine GABA from the CSF microenvironment or produce *de novo* GABA intracellularly. GABA is synthesized through the enzyme glutamate decarboxylase, which has 2 main isoforms: GAD1 and GAD2 (Grone and Maruska, 2016). Results showed that GAD2 expression was significantly upregulated in both D425 and CHLA-01R-MED cells when cultured in aCSF medium (Figure S4N), indicating *de novo* GABA production. However, paracrine GABA utilization cannot be dis-regarded, and further studies are necessary to elucidate this.

### Increased ABAT expression leads to reduced H3K4ac through HDAC3-mediated histone deacetylation

**Figure 6.**
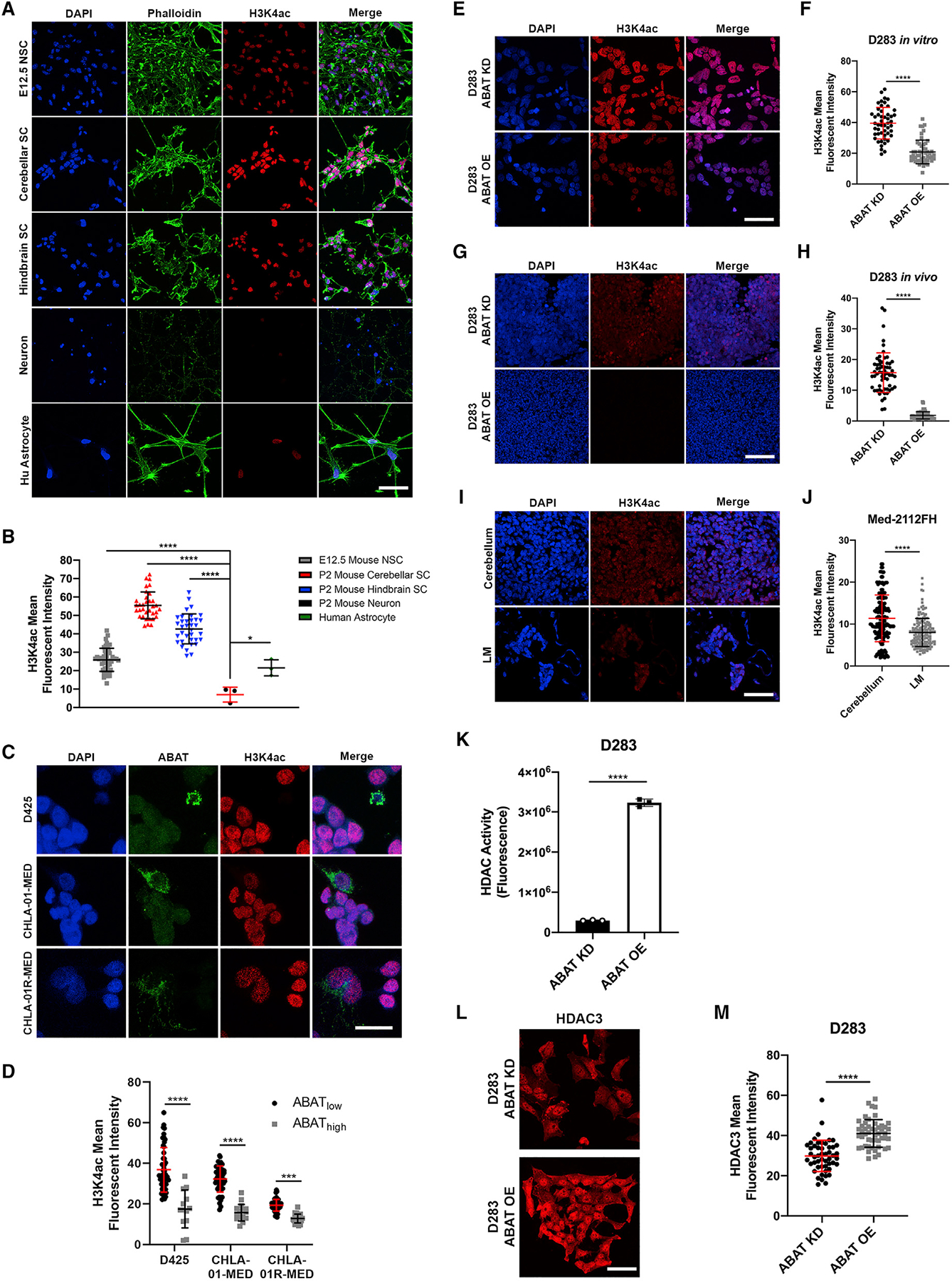
Increased ABAT expression leads to reduced H3K4ac through HDAC3-mediated histone deacetylation (A and B) H3K4ac expression analysis in neural cells depicts significantly decreased levels in neurons (n = 3) compared with E12.5 NSC (n = 48), P2 cerebellar SC (n = 34), P2 hindbrain SC (n = 35), and astrocytes (n = 3). Scale bar, 50 μm; one-way ANOVA followed by Tukey’s multiple comparison test. (C and D) ABAT_high_ cells in D425 (n = 13), CHLA-01-MED (n = 17), and CHLA-01R-MED (n = 27) display significantly decreased H3K4ac expression compared with their ABAT_low_ counterparts (n = 50 for all cell lines). Scale bar, 20 μm; t test. (E–H) ABAT OE cells in both *in vitro* (n = 50) and *in vivo* (n = 60) conditions have significantly lower H3K4ac expression compared with ABAT KD cells (n = 50 *in vitro* and n = 60 *in vivo*). Scale bar, 50 μm; t test. (I and J) LMs show significantly decreased H3K4ac (n = 160) compared with cerebellar tumors (n = 160). Scale bar, 50 μm; t test. (K) Histone deacetylase (HDAC) activity analysis reveals significantly increased HDAC activity in ABAT OE cells (n = 3). (L and M) H3K4ac deacetylase HDAC3 is significantly higher in ABAT OE cells (n = 50) compared with ABAT KD cells (n = 50). Scale bar, 50 μm; t test. n = nuclei (B, D, F, H, and J) or cells quantified (M) or replicates (K). Histogram data depict mean ± SD. *p < 0.05, **p < 0.01, ***p < 0.001, ****p < 0.0001. See also Figure S5.

ABAT expression increase in MB induces slower proliferation, the capability to metabolize GABA, and a more OXPHOS metabolic phenotype, hallmarks of differentiated neural cells. When clustering normal and MB cells by ABAT expression and mitochondrial localization, ABAT_high_ MB cells clustered with neurons (Figure S5A). Thus, we sought to interrogate whether other mechanisms in ABAT-expressing MB cells mirrored those seen in neurons. We hypothesized that alterations in chromatin structure would be present in ABAT_high_ MB cells, considering that global gene expression is found to be lower in neurons compared with stem cells (Efroni et al., 2008), and gene expression is regulated by histone modifications, with histone acetylation specifically signifying active gene expression (Verdone et al., 2005). Therefore, we interrogated the contribution of ABAT to histone 3 lysine 4 acetylation (H3K4ac) expression, an active gene promoter marker (Guillemette et al., 2011). Results showed that neurons display significantly lower H3K4ac levels compared with neural stem cells (Figures 6A and 6B). Likewise, ABAT_high_ MB cells display lower H3K4ac levels compared with ABAT_low_ cells (Figures 6C and 6D). To validate the contribution of ABAT in reducing H3K4ac levels, we analyzed H3K4ac expression *in vitro* and in xenograft models. Both D283 and D425 ABAT OE cells *in vitro* had a significant H3K4ac reduction compared with ABAT KD cells (Figures 6E and 6F; Figure S5B). Similarly, xenografted cerebellar ABAT OE cells (Figures 6G and 6H) and LMD (Figures 6I and 6J) display a significant H3K4ac reduction. To identify a potential mechanism mediating H3K4 deacetylation, we analyzed HDAC activity. Results showed that ABAT OE cells display a significant increase in HDAC activity compared with ABAT KD cells (Figure 6K; Figure S5C). H3K4ac deacetylation is mediated by HDAC3 (Phiel et al., 2001); therefore, we analyzed its expression in ABAT KD and OE cells. Results showed a significant HDAC3 upregulation in MB ABAT OE (Figures 6L and 6M; Figures S5D and S5E). We also interrogated HDAC4, HDAC11, and SIRT2, which belong to other HDAC classes. We observed that ABAT OE cells display significant increases in these proteins, suggesting other histone acetylation markers may be affected in ABAT OE cells (Figures S5F and S5G). To determine reduced H3K4ac expression function in MB LMD, we conditioned D425 cells to aCSF with or without the HDAC inhibitor trichostatin A (TSA) at concentrations known to induce HDAC inhibition without causing cell death (Vibhakar et al., 2007). We found that cells treated with TSA display decreased survival capabilities when adapting to a nutrient-depleted condition (Figure S5H), suggesting increased HDAC activity in MB cells allows easier adaptation to an environment with reduced metabolic molecules. Overall, we provide evidence indicating that MB cells with increased ABAT expression have decreased H3K4ac expression, which mirrors the expression pattern found in neurons.

## DISCUSSION

LMD is the principal cause of mortality in patients with MB; thus, discerning its biological processes is essential to improving patient outcomes. Recently, studies analyzing bulk MB tumors have identified cell populations whose genetic architectures mirror non-tumorigenic neural cells in various differentiation states (Vladoiu et al., 2019; Jessa et al., 2019; Hovestadt et al., 2019). Therefore, we hypothesized that metabolic diversity must also be present, considering that developing neural cells undergo metabolic changes as they differentiate (Zheng et al., 2016). Because metastatic cancer cells use GABA as a metabolite in a novel TME and metastatic MB tumors G3 and G4 display neuronal transcriptional profiles, we considered whether disseminated MB cells would exploit GABA metabolism to thrive in the CSF and form LMD.

To study this, we first interrogated ABAT expression in healthy tissues. Previous studies showed that ABAT mediates the GABA metabolic pathway, a bypass mechanism in the tricarboxylic acid cycle (TCAC), producing succinate and NADH, intermediates used in OXPHOS (Olsen and Delorey, 1999; Baxter, 1970). In neurodevelopment, there is increased ABAT activity in postnatal neurons compared with neural progenitors (Schousboe et al., 1989). Furthermore, rapidly dividing neural precursors shift from a glycolytic metabolic profile to an OXPHOS-dependent energetic state as they develop and become quiescent (Zheng et al., 2016), indicating that GABA metabolism and ABAT expression may have increased utilization in differentiated cells. Our work corroborated these findings, showing higher ABAT expression and mitochondrial localization in differentiated neurons and astrocytes, increased OXPHOS phenotype in neurons compared with stem cells, and an association of *ABAT* expression with increased differentiation.

Conversely, our results showed that we could not use *ABAT* as a marker to identify differentiation state in primary MB tumors. However, we noticed that although ABAT expression is decreased in MB compared with normal cerebellum, rare ABAT-expressing cells are found among the tumor bulk and less aggressive MB subtypes WNT and SHH express higher *ABAT* levels. Cell-cycle analysis, proliferation assays, and xenograft experiments showed a reduction in growth potential in cells overexpressing ABAT. Moreover, we found that low *ABAT* levels result from epigenetic silencing rather than a mutation at the gene locus, signifying that MB cells wish to retain the ability to express this gene. Based on this evidence, we questioned why a tumor whose identity is intertwined with uncontrollable growth would maintain the capacity to express *ABAT*, a protein that halts its proliferative capabilities.

Disseminated cancer cells display a dormant phenotype (Wells et al., 2013; Giancotti, 2013) and must overcome many obstacles to successfully metastasize, including satisfying their bioenergetic requirements while isolated from nutrient-supplying vasculature. As they metastasize, tumor cells lose their consistent supply of glucose and become deprived of energy (Weber, 2016), forcing them to become metabolically plastic to fulfill their energetic demands (Gouirand et al., 2018; Pavlova and Thompson, 2016). ABAT has increased function in nutrient-deprived states in plants and bacteria (Araújo et al., 2010; Feehily and Karatzas, 2013). The enzymes metabolizing GABA in both eukaryotes and bacteria are evolutionarily conserved (Michaeli and Fromm, 2015), indicating that their functions in these models may reflect similar functions in humans. Therefore, we hypothesized that MB cells maintain the ability to express ABAT to survive in the nutrient-deprived CSF and facilitate LMD.

Using metabolite concentrations from CSF of patients with MB (Benoist et al., 2003; el-Yazigi and Al-Mefty, 1985; Goldsmith et al., 1987) and considering that the CSF microenvironment is deprived of glucose in patients with LMD (Bruna et al., 2009), we derived an artificial medium to recapitulate the CSF metabolic environment. Our studies showed increased ABAT expression in cells cultured in this medium and decreased proliferation, which was expected based on our previous results. More importantly, we found that increasing the aCSF concentration in the medium results in a stepwise *ABAT* expression increase. Due to the absence of cell death in the 0%–75% aCSF conditions, we concluded that rare ABAT_high_ cells are not the only ones that can express ABAT; rather, nutrient reduction in the TME can dictate that ABAT_low_ cells increase ABAT expression to survive. To test these findings *in vivo*, we first transplanted MB cells into both the cerebellum and the leptomeningeal space. We found that ABAT expression is significantly increased in LMD, accompanied by a reduction in proliferative capabilities. Moreover, xenografted ABAT OE cells excel at seeding the leptomeningeal space, whereas ABAT KD cells do not, indicating that ABAT is necessary for LMD.

We then interrogated the mechanism governing ABAT-mediated MB cell survival in LMD. Although ABAT has been shown to be implicated in mitochondrial nucleoside metabolism, respiratory capacity, membrane potential, and mitochondrial DNA maintenance (Besse et al., 2015, 2016), we interrogated the metabolic contribution of ABAT in MB. We found that ABAT-expressing cells are capable of metabolizing GABA; however, this leads to no changes in proliferation. Instead, ABAT promotes survival in nutrient-deprived conditions, providing evidence as to why increased expression is found in the LMD compared with the primary tumor. With the metabolic similarities we observed between differentiated neurons and metastatic MB cells, we questioned whether they share similar redox states. Stem and cancer cells prefer to use glycolysis (Ito and Suda, 2014; Gu et al., 2016; Yu et al., 2017), whereas differentiated cells prefer OXPHOS (Lange et al., 2016; Beckervordersandforth et al., 2017). We found that ABAT OE increases OXPHOS. Moreover, our studies showed that MB cells cultured in aCSF display high levels of OXPHOS, similar to levels seen in neurons. These data corroborate evidence in the literature that suggests metastatic cells in the brain upregulate OXPHOS-related genes (Faubert et al., 2020).

Lastly, we compared the expression of the epigenetic modifier H3K4ac between control and ABAT OE cells. Changes in the TME induce alterations in the metabolic state of MB cells. Metabolic reprogramming in tumor cells can preclude epigenetic effects (McDonald et al., 2017; Gouirand et al., 2018) and influence cellular differentiation status (Ward and Thompson, 2012), indicating that an increase in ABAT expression, and thus a phenotypic change in metabolism, may induce transcriptional and chromatin changes in tumor cells. In both normal tissues and MB, higher ABAT expression leads to decreased H3K4ac. To deduce a mechanism for this finding, we interrogated HDAC activity and found increased HDAC activity in ABAT OE cells and increased HDAC3 expression, which preferentially deacetylates histone 3 lysine 4 (Phiel et al., 2001). Lastly, we questioned the role of deacetylation in MB cell survival in aCSF. Our results showed that by inhibiting HDAC activity with TSA, we see a significant decrease in viable cells. We hypothesized that because ABAT-mediated GABA metabolism (part of the tricarboxylic acid cycle) and OXPHOS are increased in aCSF conditions, acetylcoenzyme A (CoA) becomes more important as a metabolic mediator, rather than as an epigenetic modifier. This phenomenon is present in other cancer cells, where during nutrient starvation, acetyl-CoA is more likely to be oxidized in the mitochondria for ATP synthesis, rather than histone acetylation (Shi and Tu, 2015).

Altogether, we provide evidence that ABAT is vital for MB cell survival in the CSF and eventual LMD formation. Our data show that MB cells with increased ABAT expression mirror metabolic and epigenetic characteristics of differentiated neurons and how the TME facilitates these changes. As such, our study elucidates the importance of drawing conclusions from mechanisms in neurodevelopment to understand how these pathways can be exploited in MB.

## STAR★METHODS

### RESOURCE AVAILABILITY

#### Lead contact

Further information and requests for resources and reagents should be directed to and will be fulfilled by the Lead Contact, Josh Neman, yebrahim@usc.edu.

#### Materials availability

This study did not generate unique reagents or materials.

#### Data and code availability

Data will be made available upon request. No new code was generated in this study.

### EXPERIMENTAL MODEL AND SUBJECT DETAILS

#### Animals

Animal experiments were performed on 9–10 week old female athymic nude mice, housed in ventilated cages (3–5 animals per cage) in accordance with University of Southern California Department of Animal Resources regulations. Animals were housed in an animal room designated for immuno-deficient mice, along with all cages, bedding, and feed being sterilized to ensure a pathogen-free environment. Animals were monitored by a veterinarian and determined to be healthy and pain-free before and after experiments were performed. Animals were not used in other procedures before transplantation surgeries. All protocols for mouse experiments were approved by the Institutional Animal Care and Use Committee at the University of Southern California. All animals were humanely euthanized either when reaching a specific time point or upon presentation of signs of morbidity, including development of tumor symptoms (paralysis, hydrocephalus, weight loss, tilted head).

#### Cell Cultures

MB cell lines used in this study were from 3 of the MB subgroups: SHH, Group 3, and Group 4. UW228-2 cells are from the SHH subgroup. D283, D425, and MED-2112FH cells are from the Group 3 subgroup. CHLA-01-MED and CHLA-01R-MED cells are from the Group 4 subgroup.

Cell cultures were maintained in humidified incubators at 37°C and 5%/95% CO_2_/air atmosphere. Cells were negative for mycoplasma and were frequently checked utilizing MycoAlert Kits (VWR, Cat#75860-360) to ensure contamination-free culture conditions. Mouse neurons were isolated from postnatal day 2 mice utilizing the Worthington Biochemical Corporation Papain Dissociation System (Worthington Biochemical, Cat#LK003150) and according to manufacturer protocol. Neurons were cultured on poly-D-lysine (Sigma, Cat#P7280-5MG) coated plates and in Neuronal medium (Neurobasal-A (Life Technologies, Cat#10888022), 1 x Glutamax (ThermoFisher Scientific, Cat#35050061), 1 x B-27 Supplement (ThermoFisher Scientific, Cat#17504044), 1 x Antibiotic-Antimycotic (Life Technologies, Cat#15240062)) with half of the medium being replaced every 2 days. Medium was replaced by careful aspiration to ensure neurons were always submerged in medium. Pure neurons cultures were obtained by treating derived cells with cytosine arabinoside to eliminate other cell types. Embryonic day 12.5 neural stem cells and postnatal day 2 cerebellar and hindbrain stem cells were derived as previously described (Deshpande et al., 2019). The cerebellum and hindbrain were dissected separately to isolate stem cells endemic to each location. Mouse stem cells and human medulloblastoma cell lines CHLA-01-MED and CHLA-01R-MED were cultured on poly-ornithine (Sigma, Cat#P2533-100MG) and fibronectin (ThermoFisher Scientific, Cat#PHE0023) coated tissue culture plates in stem cell medium (Advanced DMEM/F12 (Life Technologies, Cat#126340), 1.25 x Glutamax, 0.5 x B27 Supplement, 1 x Antibiotic-Antimycotic, 20ng/mL EGF (Peprotech, Cat#AF-100-15) and 20ng/mL FGF (Peprotech, Cat#100-18B) (EGF and FGF added to medium every 3 days)). Patient-derived xenograft tissue culture cell line Med-2112FH was also cultured in stem cell medium and in sphere-forming conditions (the culture plate was placed on a rotational plate shaker inside of the incubator). Human astrocytes and medulloblastoma cell lines UW228-2, D283, and D425 were cultured on rat-tail collagen (Life Technologies, Cat#A1048301) coated tissue culture plates in glutamine-supplemented medium (Advanced DMEM/F12, 10% fetal bovine serum (Omega Scientific, Cat#FB-02), 1 x Glutamax, 1 x Antibiotic-Antimycotic). Human medulloblastoma D425 and CHLA-01R-MED cells exposed to artificial CSF medium (DMEM (no glucose, no glutamine, no phenol red, Life Technologies, Cat#A1443001), 5% fetal bovine serum, 0.25 x Glutamax, 150 μM sodium pyruvate (Life Technologies, Cat#11360-070), 0.2μM γ-aminobutyric acid (Sigma, Cat#A2129-100G), 1 x Antibiotic-Antimycotic) were cultured in rat-tail collagen and poly-ornithine/fibronectin coated tissue culture flasks, respectively. We recapitulated the CSF microenvironment by utilizing metabolite concentrations derived from CSF of patients with MB (Benoist et al., 2003; el-Yazigi and Al-Mefty, 1985; Goldsmith et al., 1987). Medulloblastoma cells were acclimated to artificial CSF gradually (complete medium %/artificial CSF medium %: 1^st^ day – 100/0, 2^nd^ day – 75/25, 3^rd^ day – 50/50, 4^th^ day – 25/75, 5^th^ day – 0/100) and medium was fully replaced every 2 days.

### METHOD DETAILS

#### Tissue Culture Plate Coating

For rat-tail collagen tissue culture flask coating, collagen (Life Technologies, Cat#A1048301) was diluted to 100μg/mL using a 0.02M acetic acid and sterile water mixture. This solution was mixed to ensure homogeneity and added to plates inside the tissue culture hood for a minimum of 2 hours. Collagen mixture was then aspirated and plates were washed with equal volume of sterile DPBS. Plates were either used immediately or stored at 4°C for future use. Poly-ornithine (Sigma, Cat#P2533-100MG) and fibronectin (ThermoFisher Scientific, Cat#PHE0023) coating was performed as previously described (Deshpande et al., 2019). For poly-D-lysine (PDL) (Sigma, Cat#P7280-5MG) coating, 1 x stock of PDL was made by adding 333.35mL of sterile ddH_2_O to 50 mg of PDL and filtered through 0.22 μm filter. Solution was added to plates and incubated at a 37°C incubator overnight. Plates were then washed twice with sterile ddH_2_O and allowed to completely dry in the tissue culture hood. Plates were then used immediately or stored at 4°C for future use.

#### Exogenous GABA Treatment

To measure the alterations in GABA Shunt mediators mRNA and differentiate GABA accumulation in ABAT_high_ and ABAT_low_ medulloblastoma cells, cells cultured in glutamine-supplemented medium were treated with exogenous GABA (Sigma, Cat# A2129-100G) at 2mM for 2 weeks. Half of the medium was replaced every 3–4 days.

#### Lentivirus Particle Production, Transduction, and Selection

*ABAT* knockdown (Sigma, Cat# SHCLNG-NM_000663) and overexpressing (GeneCopoeia, Cat#EX-A3225-Lv130) lentiviral particles were produced using the 293T cell system. 24 hours prior to transfection, 4.0×10^6^ 293T cells were plated in a T75 tissue culture flask in glutamine-supplemented medium. The following day, 7μg of DNA vector, 3μg Gag/Pol, 1.45μg Rev, and 1.75μg VSV-G were diluted in 1mL serum-free Advanced DMEM/F12 (Life Technologies, Cat#126340). PEI (Polysciences, Cat#23966-2) was then added to the diluted DNA mixture at a 3:1 ratio (PEI (μL) and total DNA (μg)) and immediately vortexed. The DNA and PEI mixture was incubated at room temperature for 15 minutes. The mixture was then mixed with 9mL of glutamine-supplemented medium and added to the 293T cell flask. Cells were culture for 60 hours, after which the supernatant was collected and centrifuged to remove cell debris. The supernatant was then either used immediately or aliquoted and stored at −80°C for future use.

For transduction and selection, human medulloblastoma cells D283 and D425 were plated at 1.0×10^5^ cells/well in a collagen coated 6-well tissue culture dishes. Lentivirus containing supernatant was added to the well in conjunction with 10μg/mL polybrene. Cells were incubated for 48 hours after which the medium was replaced with glutamine-supplemented medium. After 72 hours, cells were treated with 1μg/mL puromycin to select for transduced cells. A non-transduced control well was also treated with puromycin to ensure proper cell selection. 5 *ABAT* KD variants were queried and 1 variant with significant knockdown was chosen to be used in experiments.

#### Cell Cytotoxicity Analysis

To analyze the effects of ABAT KD or ABAT OE on the cytotoxic effects of cisplatin (Sigma, Cat#232120-50MG), vincristine (Selleck Chemicals, Cat#S1241), and NEO216 (provided by Neonc Technologies) in medulloblastoma cells, we first deduced the IC_50_ of these drugs in D283 and D425 Scrambled cell lines. 1×10^4^ cells were plated into each well in a collagen-coated 96-well black plate with a clear bottom (Greiner Bio-one, Cat#655090). Cells were allowed to settle for 24 hours. Afterward, cells were treated with logarithmically increasing concentrations of either cisplatin (10nM-100μM) or vincristine (1nM-1μM). Each concentration was administered in triplicate. Cytotoxicity was determined using the LIVE/DEAD Viability/Cytotoxicity Kit (ThermoFisher Scientific, Cat#L3224). Once the IC_50_ was determined, D283 and D425 Scrambled, ABAT KD, and ABAT OE cells were plated in identical plates in triplicate and treated with the IC_50_ values for both cisplatin and vincristine.

#### Xenograft Models in Athymic Nude Mice

Three medulloblastoma xenograft models were established: (1) Primary tumor models with 2×10^5^ D283 Scrambled, ABAT KD, and ABAT OE medulloblastoma cells transplanted into the cerebellum, (2) Competitive Leptomeningeal models with 1×10^5^ D283 ABAT KD and 1×10^5^ ABAT OE medulloblastoma cells transplanted into the cisterna magna, and (3) A clinically relevant dual-injection model depicting primary and metastatic medulloblastoma with 1×10^5^ Med-2112FH injected into both the cerebellum and the cisterna magna. Mice were initially anesthetized under 5% isoflurane (Vetone, Cat#502017) and maintained at 2%–2.5% isoflurane. To model primary disease, tumor cells were injected into the cerebellum utilizing a stereotaxic frame. Cells were transplanted at −6.47 Bregma, 1mm lateral of the sagittal suture, and 1mm into the cerebellum. To model leptomeningeal disease, surgery set-up mirrored cerebellar injections, however, cells were injected into the cisterna magna as previously shown (Xavier et al., 2018). All transplanted lines expressed luciferase for *in vivo* bioluminescent imaging (BLI) post-transplantation. BLI was performed 3 days and 7 days post-injection, and once every week afterward for the single-injection models. Mice were monitored for presentation of tumor burden related symptoms and humanely euthanized. For the dual-injection models, BLI was performed 3 days post-injection, and every 3–4 days afterward. Mice were euthanized 21 days post-transplantation to observe immediate effects of the leptomeningeal microenvironment on medulloblastoma cells.

For the cerebellar and leptomeningeal models utilizing the D283 Scrambled, ABAT KD, and ABAT OE cell lines, confirmation of ABAT knockdown or overexpression was validated 2 days prior to transplantation surgery.

Bioluminescent imaging scales for the D283 Scrambled, ABAT KD, and ABAT OE cerebellar xenograft model differ due to the fact that normalization of the imaging scales did not show the presence of any tumor at any time in the ABAT OE group. Thus, to show the presence and subsequent decrease of the ABAT OE tumor, we increased the imaging scale sensitivity.

#### DNA Methylation Analysis

DNA methylation sequences from 104 medulloblastoma tissues were obtained from Northcott et al. (2017) from their published data. Among those 104 tumor samples, there are 26 samples each in the WNT, SHH, Group 3, and Group 4 subgroups. DNA methylation data from 26 normal cerebellar tissues was obtained from Gene Expression Omnibus by Stanley Brain Bank (GEO: GSE137223). Causes of death include suicide, cardiac failure or other accidents as determined by forensic pathologists (Policicchio et al., 2020). DNA methylation was measured using Illumina HumanMethylation450K BeadChip platform. Untransformed methylation β values were used for analysis and comparison. For occasional missing values from these two sources, imputation was done with *impute.knn* function from the package ‘*impute’* (version 1.58.0) in R 3.6.0 with default parameters. CpGs from the promoter regions of *ABAT* and *ALDH5A1* were filtered out based on Illumina’s v1.2 annotation with the package ‘*IlluminaHumanMethylation450kan-no.ilmn12.hg19’* (version 0.6.0) in R 3.6.0.

#### BrdU Cell Proliferation ELISA, HDAC Activity, and Intracellular Succinate Assays

To quantify cell proliferation, the BrdU Cell Proliferation ELISA Kit (Abcam, Cat#ab126556) was used. Medulloblastoma cells were plated into: (1) for D283 and D425 MB cells, a collagen-coated and (2) for CHLA-01R-MED, a poly-ornithine/fibronectin, 96-well black plate with a clear bottom (Greiner Bio-one, Cat#655090). 1,000 D283 and D425 cells and 1×10^4^ CHLA-01R-MED cells were plated into each well. Cells were then allowed to settle for 24 hours. To measure the effect of artificial CSF on cell proliferation, medium was replaced and the experiment was allowed to continue for 6 additional days, with medium being replaced on Day 3. To measure the effect of increasing GABA concentration on cell proliferation, all media was removed and replaced with either 0, 2μM, 200 μM, or 2mM GABA supplemented medium. The experiment was then allowed to continue for 6 days, with the entirety of medium and GABA supplementation being refreshed on Day 3. For both experiments, on Day 5, the BrdU reagent was added for incorporation into replicating cells for 24 hours, in line with manufacturer specifications. At the end of the time point, the plate was then processed according to manufacturer protocol.

For HDAC activity analysis, the HDAC-Glo^™^ I/II Assays and Screening System (Promega, Cat#G6420) was used. 1,000 D283 and D425 Scrambled, ABAT KD, and ABAT OE cells were plated into a collagen-coated 96-well black plate with a clear bottom. Cells were allowed to settle for 24 hours, after which medium was replaced and the experiment was allowed to continue for 6 additional days. On Day 3, the entirety of the medium was replaced. At the end of the time point, the plate was then processed according to manufacturer protocol.

To measure GABA metabolism capabilities in medulloblastoma cells after GABA treatment, we measured intracellular succinate utilizing the Succinate Assay Kit (Abcam, Cat#ab204718). 2×10^6^ cells were plated into a T25 flask which was coated with either collagen (for D283, D425) or with poly-ornithine/fibronectin (for CHLA-01R-MED). Medulloblastoma cells were then allowed to stabilize for 3 days. Afterward, cells were treated with 2mM of exogenous GABA for 24 hours. 1×10^6^ cells were then collected and analyzed according to manufacturer specifications.

#### Histology, Immunofluorescence, Immunocytochemistry

Medulloblastoma tissue samples were obtained from US Biomax, Inc. (Cat#BC17012c). The medulloblastoma tissue array contained 60 medulloblastoma punch biopsies (3 tissue samples from 20 patients), and normal cortical and cerebellar controls which were paraffin embedded. Patient samples histological subtypes were then determined by a neuropathologist. For mouse brain and spine samples, mice were perfused first with DPBS and then with 4% formaldehyde. Tissues were extracted and placed in 4% formaldehyde for 1 week. Samples were then processed by the USC Department of Pathology.

Immunofluorescence staining was performed on the tissues based on previous established protocol (Neman et al., 2014). In short, paraffin-embedded samples were stained by first dewaxing the tissue with xylene (3x, 5 minutes each) and then hydrating them with an alcohol gradient (2× 100% ethanol, 2× 95%, 1× 70%, and 1x DPBS, 5 minutes each). Antigen recovery was performed by treating tissues with Na-citrate buffer (10mM, pH 6.0) at 85–90°C for 30 minutes and 10 minutes on the bench to cool. Slides were then washed in DPBS for 5 minutes and permeabilized with 0.3% Triton X-100 in DPBS for 45 minutes at 37°C. Slides were then washed in DPBS (3x, 5 minutes each). To block non-specific binding sites, tissues were incubated in a 1:1 mixture of SEA BLOCK Blocking Buffer (ThermoFisher Scientific, Cat#37527) and DPBS with 0.3M Glycine at room temperature for 1 hour. Tissues were stained with primary antibody in the same blocking buffer overnight at 4°C. Samples were then washed with DPBS (3x, 5 minutes each) and stained in secondary antibody (in PBS) for 1 hour at room temperature (in the dark). Slides were again washed with DPBS (3x, 5 minutes each) and mounted with ProLong^™^ Gold Antifade Mountant with DAPI (ThermoFisher Scientific, Cat#P36931). Coverslips were sealed with nail polish and allowed to dry for 24 hours before being stored in a slide box at 4°C.

Immunocytochemical was performed on medulloblastoma cell samples plated on collagen or poly-ornithine/fibronectin coated 15mm coverslips. Cells were initially plated at a density of 5×10^5^ cells/well and allowed to grow for 3 days before being fixed in 4% formaldehyde for 10 minutes. Immunocytochemical staining was then carried out following our established protocol (Deshpande et al., 2019). In short, coverslips were permeabilized with 0.3% Triton X-100 in DPBS for 30 minutes at 37°C. Samples were then washed in DPBS once for 5 minutes and then non-specific binding sites were blocked in a 1:1 mixture of SEA BLOCK (ThermoFisher Scientific, Cat#37527) and DPBS for 1 hour. Primary antibody in the blocking buffer was then added and incubated for overnight at 4°C. Coverslips were washed with DPBS (3x, 5 minutes each) and incubated with secondary antibody (in PBS) for 1 hour at room temperature (in the dark). Coverslips were then washed with DPBS (4x, 5 minutes each) and mounted onto Superfrostâ Plus Micro Slides (VWR, Cat#48311-703) and sealed with nail polish. Slides were then allowed to rest for 24 hours before being stored in a slide box at 4°C.

Mouse-derived human mitochondrial antibody was stained on mouse tissue sections using the M.O.M. (Mouse on Mouse) Blocking Reagent (Vector Laboratories, Cat#MKB-2213). For quantification purposes, control samples were staining in the same manner, excluding administration of the primary antibody. This was done to determine the background staining levels of the secondary antibody. Primary and secondary antibodies used for staining can be found in the Key Resources Table.

#### Real-time Quantitative PCR Analysis

qPCR was carried out based on previous established protocol (Deshpande et al., 2019). Primer list can be found in Table S1. In short, cells were harvested from culture and spun down using a centrifuge. Cells were either immediately processed or stored at −80°C for future analysis. mRNA was extracted using the RNeasy Plus Mini Kit (QIAGEN, Cat#74136) and QIAshredder Tissue and Cell Homogenizer (QIAGEN, 79656) according to manufacturer protocol. mRNA concentration was measured using the Varioskan^™^ Lux multimode microplate reader (ThermoFisher Scientific). For normalization, 1μg of RNA was used to make cDNA utilizing the Maxima First Strand cDNA Synthesis Kit for RT-qPCR (ThermoFisher Scientific, Cat#K1641) and according to manufacturer thermocycler guidelines. RT-qPCR was then performed at a final volume of 20μL/well using PowerUp^™^ SYBR^™^ Green Master Mix (10μL, ThermoFisher Scientific, Cat#A25778), forward and reverse primers from IDT (1μL), cDNA (10ng), and nuclease-free water. RT-qPCR reaction was performed on the Applied Biosystems QuantStudio 6 Flex Real-Time PCR System.

#### Bioinformatics and Databases

We utilized 4 main sources: (1) Allen Institute Developing Mouse Brain Atlas (https://developingmouse.brain-map.org/) and Brain Explorer 2 (https://developingmouse.brain-map.org/static/brainexplorer) (2) R2 genomics analysis and visualization platform (https://hgserver1.amc.nl/cgi-bin/r2/main.cgi) (3) Genevestigator (https://genevestigator.com/, (Hruz et al., 2008) and (4) cBioPortal for Cancer Genomics (https://www.cbioportal.org/, (Gao et al., 2013; Cerami et al., 2012).

For analysis of *ABAT* and *ALDH5A1* expression through human development, we utilized the R2 genomics analysis and visualization platform and queried the “Normal Brian Development – Brain Span – 524 – rpkm – brspv10rs” database. We specifically selected samples under the “Cerebellar cortex” or “Cerebellum” subsets.

*ABAT* and *MYC* expression analysis in histopathologically stratified medulloblastoma was done using Genevestigator. Normal brain samples were refined by selecting the “Anatomy” condition, then under the “Tissue” section selecting for “Nervous System” and “Central Nervous System” subcategories. Samples were further filtered by selecting for samples that were deemed “Healthy.” Medulloblastoma samples were chosen by selecting the “Cancer” condition, then selecting for “Neoplasms of eye/brain/central nervous system,” “Brain,” “Cerebellum,” and finally the “Cerebellum, Medulloblastoma, NOS,” “Cerebellum, Desmoplastic Medulloblastoma,” and “Cerebellum, Large Cell Medulloblastoma” subcategories.

*ABAT* expression analysis in medulloblastoma molecular subgroups was performed using the MegaSampler analysis in R2 which allowed for comparison of *ABAT* gene expression across multiple datasets. Normal cerebellar samples were used from the dataset “Normal cerebellum – Roth – 9 – MAS5.0 – u133p2” which is deposited at GEO: GSE3526. Medulloblastoma samples were combined from two datasets: “Tumor Medulloblastoma – Gilbertson – 76 – MAS5.0 – u133 p2” (GEO: GSE37418) AND “Tumor Medulloblastoma – Pfister – 223 – MAS5.0 – u133p2.”

Whole genome sequencing analysis of *ABAT* mutation status was done using cBioportal, selecting for medulloblastoma analysis, and querying for *ABAT*.

Analysis of neural subtypes containing *ABAT* in the developing mouse brain was performed as previously described (Carter et al., 2018).

Determination of differentiation status of MB cells according to ABAT expression was performed as previously described (Hovestadt et al., 2019).

#### Measuring GABA metabolic fluxes through ^13^C magnetic resonance spectroscopy

*In vitro* D283 medulloblastoma cells were plated in [1-^13^C]-glucose growth culture without the presence of GABA. After 6 hours of incubation medulloblastoma cells were then harvested for ^13^C-GABA MRS. *In vivo*, mice xenografted with D283 medulloblastoma tumors for 14 days were infused with ^13^C-glucose prior to sacrifice for 1 hour. Isolated medulloblastoma and normal brain tissues were then resected for ^13^C-GABA mass resonance spectroscopy. Mass resonance spectroscopy imaging for ^13^C-GABA was conducted accordingly to previously established protocol (Petroff and Rothman, 1998).

#### Fluorescence Associated Cell Sorting

The Cytofix/Cytoperm^™^ Fixation/Permeabilization Kit (BD Biosciences, Cat#554714) was used for fluorescence associated cell sorting analysis. 1×10^6^ cells were initially harvested and fixed in the Cytofix/Cytoperm solution for 20 minutes at 4°C. Cells were then washed with Perm/Wash (2x). Cells were incubated in primary antibody diluted in 1:1 mixture of SEA BLOCK (ThermoFisher Scientific, Cat#37527) and Perm/Wash overnight at 4°C on a mini-tube rotator. Samples were then washed with Perm/Wash (2x) and incubated in secondary antibody at room temperature for 1 hour. Samples were then washed with Perm/Wash (2x) and resuspended in ice-cold buffer (DPBS, 10% FBS, 10% Sodium-Azide). Samples were analyzed at the USC Stem Cell Flow Cytometry Facility.

#### Cell Cycle Analysis

1×10^6^ cells were collected in a 1.5mL Eppendorf tube and resuspended in glutamine-supplemented medium. Hoechst 33342 was used for cell cycle analysis in conjunction with 7-AAD for viability purposes. Cells were incubated with Hoechst 33342 (5μg/mL) for 60 minutes. 0.5 μL 7-AAD (stock solution – 20mM) was added to the tube and incubated for 5 minutes. Cells were then pelleted by centrifugation and resuspended in ice-cold buffer (DPBS, 10% FBS, 10% Sodium-Azide). Samples were analyzed at the USC Stem Cell Flow Cytometry Facility.

#### Single-cell transcriptomics

For single-cell analysis of normal cerebellar tissue (Carter et al., 2018) and MB (Vladoiu et al., 2019), analyses were conducted as previously described.

The single-cell atlas of human leptomeningeal metastasis from breast (n = 3) and lung cancer (n = 2) is deposited at GEO: GSE150660. Data were analyzed as described in and cell annotations were retrieved from Chi et al. (2020). Apoptotic cells, low-complexity cells, and cell doublets were excluded from all analyses. Please note that the PCA-reduced matrix was de-noised and missing values were imputed with MAGIC (van Dijk et al., 2018). All UMAP plots show imputed, post-MAGIC expression values. Dataset can be accessed through following link: https://www.ncbi.nlm.nih.gov/geo/query/acc.cgi?acc=GSE150660, using reviewer token: qzyfwwosltkxzuv.

#### Two-photon confocal microscopy targeting endogenous fluorophores

All cell lines analyzed were initially plated on collagen, poly-ornithine/fibronectin, or poly-D-lysine coated 35mm dishes with a 20mm diameter glass center (MatTek Life Sciences, P35G-0-20-C) and allowed 3 days to equilibrate. Cells analyzed for optical redox ratio had no further manipulation performed. Cells analyzed for increase in NADH after exogenous GABA treatment were administered 2mM exogenous GABA for 24 hours. Imaging of autofluorescent metabolites, NADH and FAD, and data analysis were carried out following our established protocol (Zhou et al., 2020).

### QUANTIFICATION AND STATISTICAL ANALYSIS

Statistical details of the experiments, including statistical test used, definition of “n” and value of “n,” and dispersion and precision measures can be found in the figure or figure legend. Please note that p values are found in the figure legends.

#### Immunohistochemistry and Immunocytochemistry Quantification

Images used for quantification were taken at 2048×2048 resolution and exported as a maximum projection image. Mean fluorescent intensity of regions of interest was quantified using ImageJ (https://imagej.net/Welcome; Schneider et al., 2012). Co-localization was determined on the LAS X microscopy software using default settings. Co-localization was performed on the maximum projection image.

#### Statistical Analysis

Results were analyzed using Graphpad Prism 8. To assess statistical significance, Student’s t test, one-way ANOVA, two-way ANOVA, and log-rank statistical analyses were used. For one-way and two-way ANOVA, post hoc analysis was performed using Tukey’s multiple comparison test where necessary. All histogram data depict individual data points with the mean ± SD (standard deviation) and p < 0.05 was considered to be statistically significant. Star significance depicted as *p < 0.05, **p < 0.01, ***p < 0.001, ****p < 0.0001.

## Supplementary Material

supplementary material

Supplementary table

## Figures and Tables

**Table:** KEY RESOURCES TABLE

REAGENT or RESOURCE	SOURCE	IDENTIFIER
Antibodies		
Rabbit ABAT antibody	Novus	Cat#: NBP2-21598
Mouse ABAT antibody	OriGene Technologies	Cat#: UM800070; RRID: AB_2629180
Rabbit ALDH5A1 antibody	Genetex	Cat#: GTX110181; RRID: AB_1949630
Mouse Mitochondria antibody	Abcam	Cat#: Ab92824; RRID: AB_10562769
Rabbit AIF antibody, Alexa Fluor 488 Conjugated	R&D Systems	Cat#: IC1457G
Rabbit GABA antibody	Genetex	Cat#: GTX125988; RRID: AB_11173015
Rabbit cMYC antibody	Cell Signaling Technologies	Cat#: 139875
Rabbit H3K4ac antibody	Abcam	Cat#: Ab176799; RRID: AB_2891335
Rabbit H3K4ac antibody	Invitrogen	Cat#: MA5-33157; RRID: AB_2811973
Rabbit Ki67 antibody	Biocare Medical	Cat#: CRM 325 A
Mouse GAD1 antibody	Chemicon International	Cat#: MAB5406; RRID: AB_2278725
Rabbit GAD2 antibody	Genetex	Cat#: GTX32615
Alexa Fluor 488 Conjugated Phalloidin	Thermo Fisher Scientific	Cat#: A12379
Rabbit HDAC3 antibody	Genetex	Cat#: GTX113303; RRID: AB_10721050
Rabbit HDAC4 antibody	Sigma	Cat#: ABE262; RRID: AB_11210266
Rabbit HDAC11 antibody	Thermo Fisher Scientific	Cat#: PA5-101007; RRID: AB_2850485
Rabbit SIRT2 antibody	Abcam	Cat#: Ab67299; RRID: AB_1142864
Cy3-conjugated Goat Anti-Rabbit secondary antibody	Jackson ImmunoResearch	Cat#: 111-165-144; RRID: AB_2338006
Alexa Fluor 488-conjugated Goat Anti-Mouse secondary antibody	Jackson ImmunoResearch	Cat#: 115-545-146; RRID: AB_2307324
Alexa Fluor 647-conjugated Goat Anti-Rabbit secondary antibody	Jackson ImmunoResearch	Cat#: 115-605-144
Bacterial and virus strains		
ABAT Overexpression - ORF expression clone for ABAT (NM_020686.5) - Purified plasmid	GeneCopoeia	Cat#: EX-A3225-Lv130
ABAT Knockdown - MISSION shRNA Bacterial Glycerol Stock	Sigma	Cat#: SHCLNG-NM_000663
Biological samples		
Medulloblastoma Tissue Array	US Biomax, Inc.	Cat#: BC17012b
Chemicals, peptides, and recombinant proteins		
Gamma-aminobutyric acid	Sigma	Cat#: A2129-100G
Cisplatin	Sigma	Cat#: 232120-50MG
Vincristine sulfate	Selleck Chemicals	Cat#: S1241
NEO216 (POH-Valproate)	Norac Pharma	Cat#: PSG-009-DAD-096
Critical commercial assays		
BrdU Proliferation Assay	Abcam	Cat#: Ab126556
Succinate Assay Kit	Abcam	Cat#: Ab204718
HDAC Glo I/II Assay	Promega	Cat#: G6420
LIVE/DEAD Viability/Cytotoxicity Kit	Thermo Fisher Scientific	Cat#: L3224
Experimental models: cell lines		
Mouse Neurons and Stem Cells	Deshpande et al., 2019	N/A
UW228-2	Laboratory of Anat Erdreich Epstein	N/A
D283	Laboratory of Anat Erdreich Epstein	N/A
D425	Laboratory of Anat Erdreich Epstein	N/A
CHLA-01-MED	ATCC	Cat#: CRL-3021
CHLA-01R-MED	ATCC	Cat#: CRL-3034
Med-2112FH	Brain Tumor Resource Lab - Fred Hutch	https://research.fredhutch.org/olson/en/btrl/order.html
Experimental models: organisms/strains		
Mouse: Athymic Nude	The Jackson Laboratory	NU/J
Oligonucleotides		
See Table S1 for Primer List		N/A
Software and algorithms		
Prism	Graphpad Software	N/A
ImageJ	Schneider et al., 2012	https://imagej.nih.gov/ij/

## Highlights

- ABAT expression increases during normal neurodevelopment
- Medulloblastoma (MB) expresses decreased ABAT compared with normal cerebellum
- ABAT-positive MB cells display neuron-like metabolic and histone characteristics
- ABAT is required for disseminated MB cells to form leptomeningeal metastases
