# Medulloblastoma uses GABA transaminase to survive in the cerebrospinal fluid microenvironment and promote leptomeningeal dissemination

**DOI:** 10.1016/j.celrep.2021.109475

**Published:** 2021-07-27

**Authors:** Vahan Martirosian, Krutika Deshpande, Hao Zhou, Keyue Shen, Kyle Smith, Paul Northcott, Michelle Lin, Vazgen Stepanosyan, Diganta Das, Jan Remsik, Danielle Isakov, Adrienne Boire, Henk De Feyter, Kyle Hurth, Shaobo Li, Joseph Wiemels, Brooke Nakamura, Ling Shao, Camelia Danilov, Thomas Chen, Josh Neman

In the originally published version of this article, there were two errors in Figure 6. In Figure 6B, the legend colors did not match the dataset, and in Figure 6I, the merged image was displayed first, instead of DAPI. The figure has been corrected online, and the original and corrected figures appear below.

The authors regret this error.

## Figures and Tables

**Figure 6.**
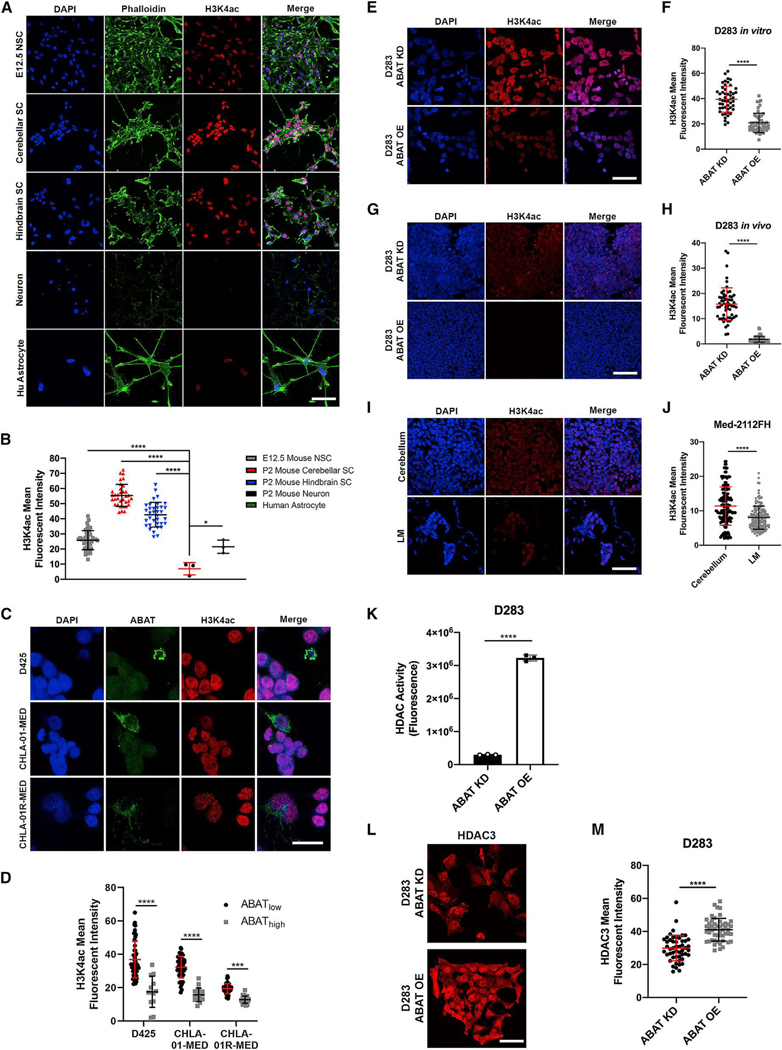
Increased ABAT expression leads to reduced H3K4ac through HDAC3-mediated histone deacetylation (corrected)

**Figure 6.**
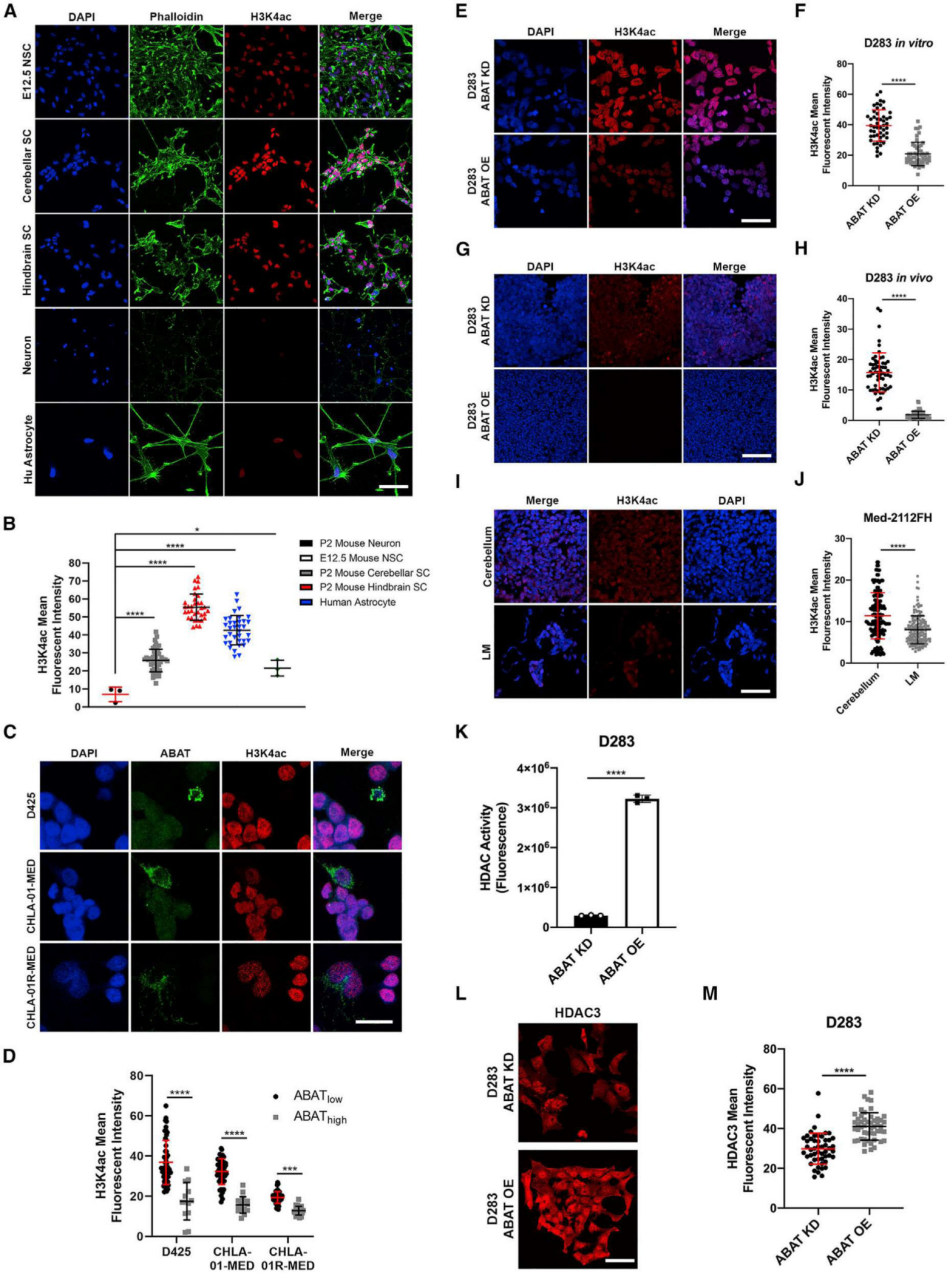
Increased ABAT expression leads to reduced H3K4ac through HDAC3-mediated histone deacetylation (original)

