# Supplementary material for: Medulloblastoma uses GABA transaminase to survive in the cerebrospinal fluid microenvironment and promote leptomeningeal dissemination
Source: Cell Rep. Author manuscript; Available in PMC 2022 Jun 29. (PMC8848833; doi:10.1016/j.celrep.2021.109302)
Supplement: supplementary material [file NIHMS1774599-supplement-supplementary_material.pdf]

Cell Reports, Volume *35*

Supplemental information

Medulloblastoma uses GABA transaminase to survive in the cerebrospinal fluid microenvironment and promote leptomeningeal dissemination

Vahan Martirosian, Krutika Deshpande, Hao Zhou, Keyue Shen, Kyle Smith, Paul Northcott, Michelle Lin, Vazgen Stepanosyan, Diganta Das, Jan Remsik, Danielle Isakov, Adrienne Boire, Henk De Feyter, Kyle Hurth, Shaobo Li, Joseph Wiemels, Brooke Nakamura, Ling Shao, Camelia Danilov, Thomas Chen, and Josh Neman

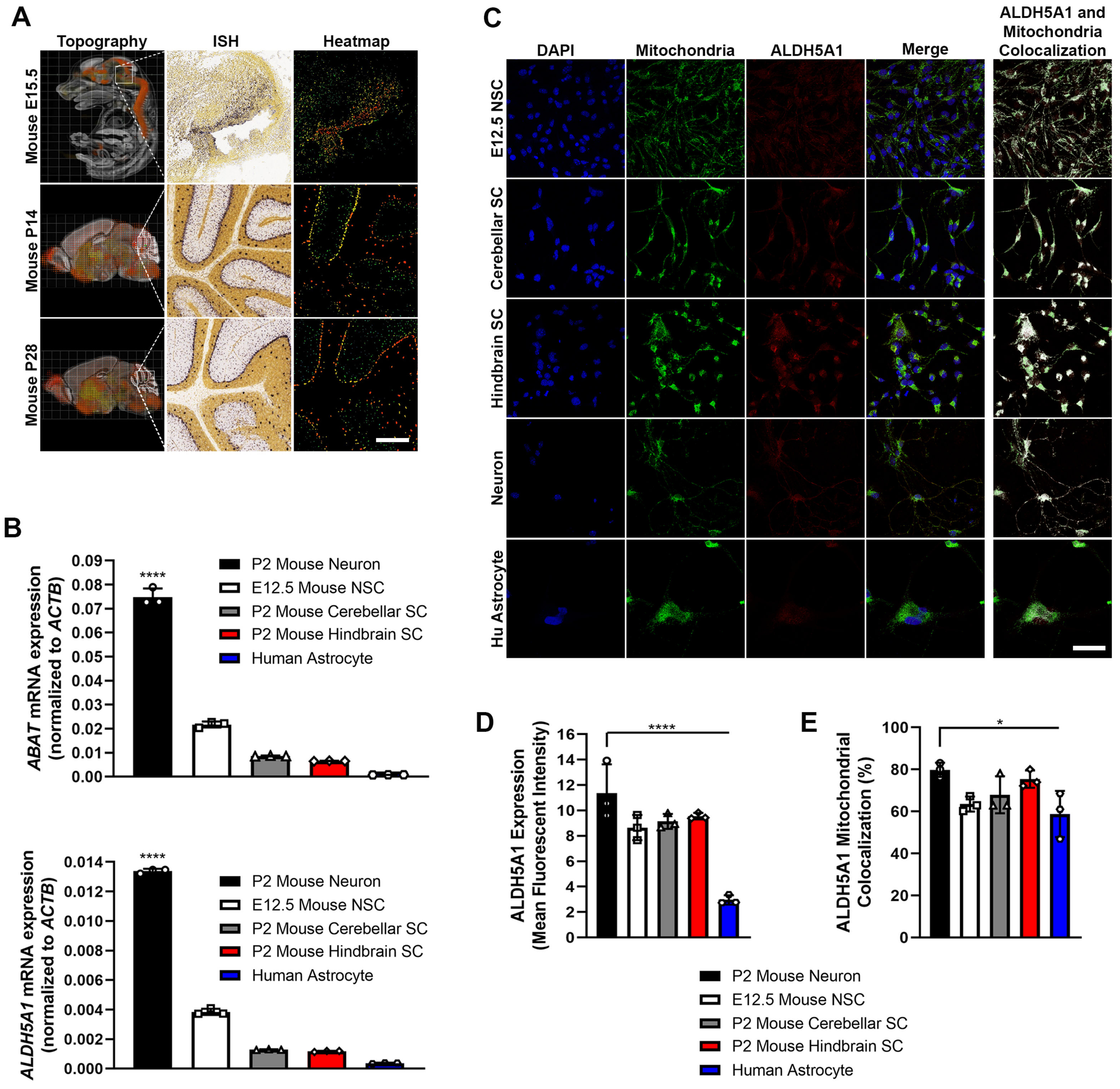

Figure S1. ABAT is localized in GABAergic neural cell mitochondria and is associated with a differentiated phenotype, Related to Figure 1. **A.** *ABAT* topography and *in situ* hybridization (ISH) data from late embryonic and early post-natal stages reveals a slow *ABAT* expression increase in the purkinje cell layer of the developing cerebellum. Scale Bar, 250µm. **B.** *ABAT* and *ALDH5A1* mRNA expression is highest in the differentiated mouse neuron. One-way ANOVA. **C-E.** ALDH5A1 expression and mitochondrial localization (n=3) is only highest in neurons compared to astrocytes. Scale bar, 50µm; One-way ANOVA. n = cells quantified. Histogram data depict mean ± SD. Star Significance: *$p<0.05$, ****$p<0.0001$. One-way ANOVA was followed by Tukey's multiple comparison test.

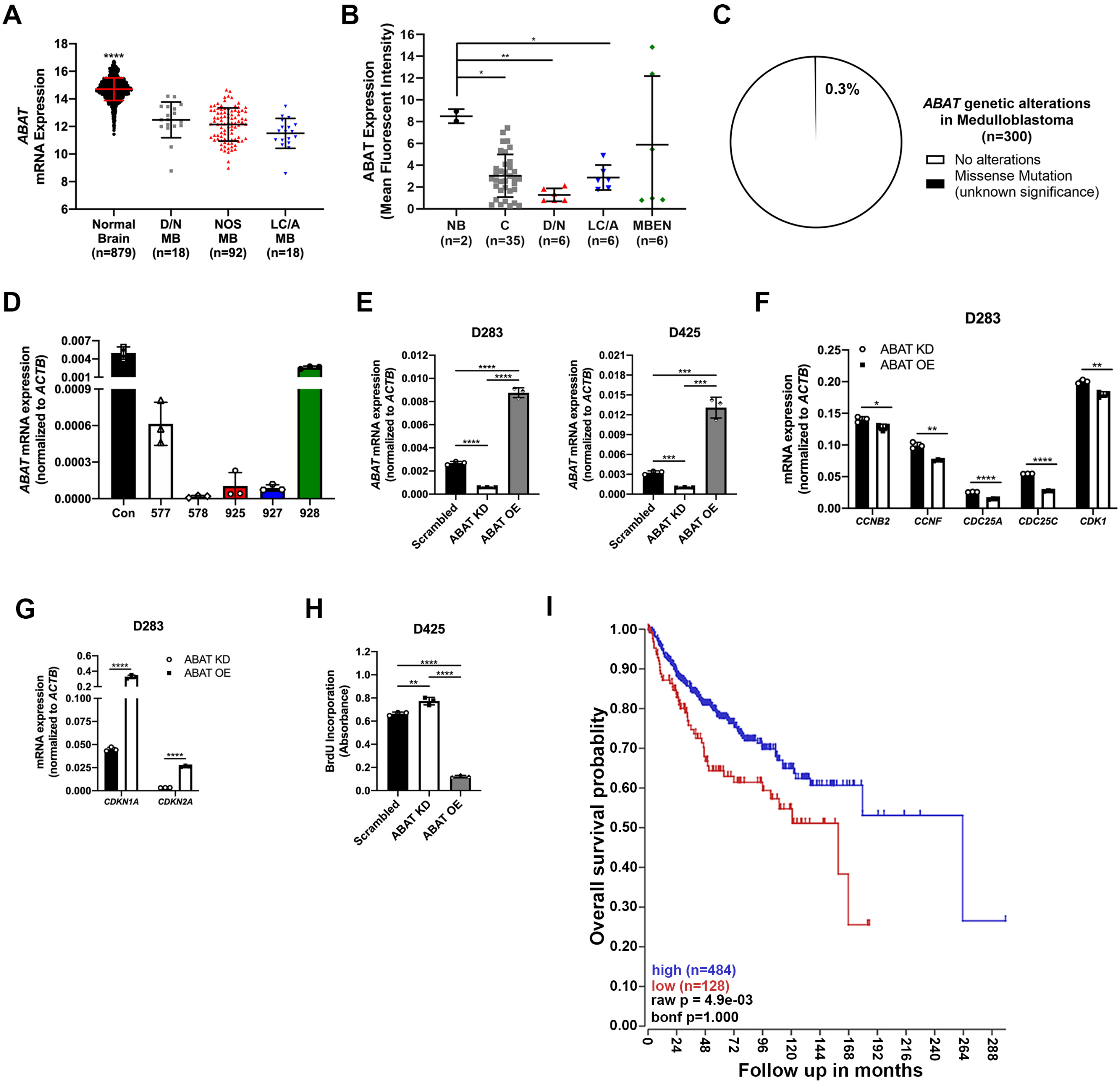

Figure S2. Higher *ABAT* expression is found in less aggressive MB subtypes and signifies a reduced proliferative potential, Related to Figure 2.

A. RNA sequencing analysis of *ABAT* mRNA levels in 879 normal brain and 128 histopathologically stratified (Hruz et al., 2008) MB samples shows decreased *ABAT* expression in MB tumors. **B.** Quantification of ABAT immunofluorescence (IF) staining in 2 normal brain and 53 MB tumor punch biopsies (US Biomax, Inc.) shows significant expression decrease in C, D/N, and LC/A MB tumors. One-way ANOVA; NB = Normal brain; C = Classic; D/N = Desmoplastic/Nodular; LC/A = Large Cell/Anaplastic. **C.** Whole genome sequencing analysis of *ABAT* in 300 MB tumors (Gao et al., 2013, Cerami et al., 2012) reveals 0.3% of tumors have a missense mutation of unknown significance. **D.** 5 *ABAT* KD variants were tested to determine a variant with significant KD. Variant 927 was used for all experiments. **E.** D283 and D425 cells transduced with *ABAT* KD and OE vectors show significant *ABAT* knockdown and overexpression, respectively. t-test. **F.** mRNA analysis of cell cycle mediators shows significant decrease in expression in ABAT OE cells. t-test. **G.** ABAT OE cells show significant increase in cell cycle inhibitors. t-test. **H.** D425 ABAT OE cells show significant reduction in proliferation rate (n=3). One-way ANOVA. **I.** Kaplan-Meier curve of MB patients expressing high (blue line) and low (red line) ABAT expression. Patients with higher ABAT levels had longer survival. n = replicates. Histogram data depict mean ± SD. Star Significance: *$p<0.05$, **$p<0.01$, ***$p<0.001$, ****$p<0.0001$. One—way ANOVAs were followed by Tukey's multiple comparison test (A, B, H).

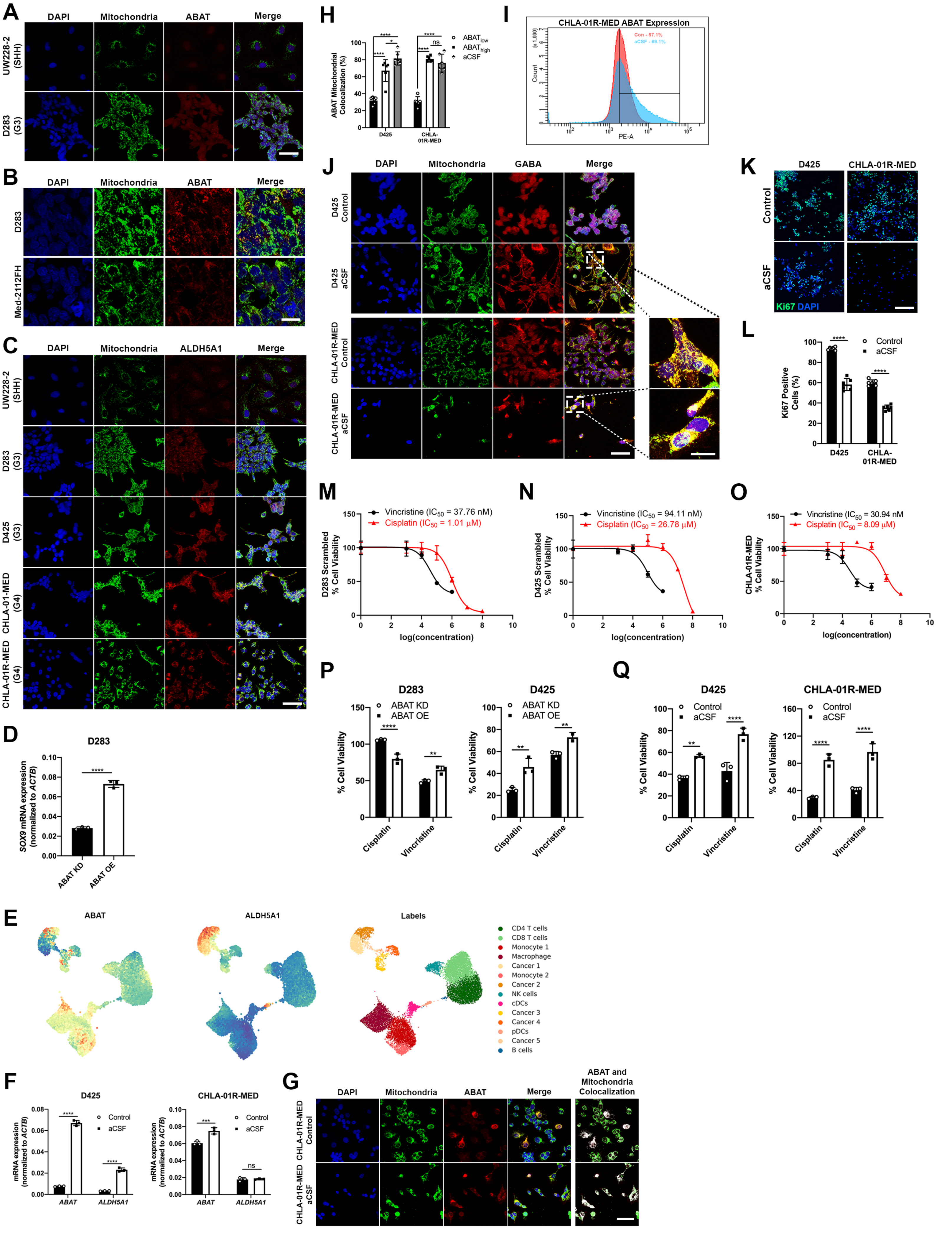

A
DAPI
Mitochondria
ABAT
Merge
UW228-2 (SHH)
D283 (G3)
B
D283
Med-2112FH
C
ALDH5A1
D425 (G3)
CHLA-01-MED (G4)
CHLA-01R-MED (G4)
D
D283
SOX9 mRNA expression (normalized to ACTB)
ABAT KD
ABAT OE
E
ABAT
ALDH5A1
Labels
CD4 T cells
CD8 T cells
Monocyte 1
Macrophage
Cancer 1
Monocyte 2
Cancer 2
NK cells
cDCs
Cancer 3
Cancer 4
pDCs
Cancer 5
B cells
F
D425
CHLA-01R-MED
mRNA expression (normalized to ACTB)
Control
aCSF
G
ABAT and Mitochondria Colocalization
CHLA-01R-MED Control
CHLA-01R-MED aCSF
H
ABAT Mitochondrial Colocalization (%)
ABATlow
ABAThigh
I
CHLA-01R-MED ABAT Expression
Con - 57.1%
aCSF - 69.1%
Count
PE-A
J
GABA
D425 Control
D425 aCSF
K
Ki67 DAPI
L
Ki67 Positive Cells (%)
M
Vincristine (IC50 = 37.76 nM)
Cisplatin (IC50 = 1.01 μM)
D283 Scrambled % Cell Viability
log(concentration)
N
Vincristine (IC50 = 94.11 nM)
Cisplatin (IC50 = 26.78 μM)
D425 Scrambled % Cell Viability
O
Vincristine (IC50 = 30.94 nM
Cisplatin (IC50 = 8.09 μM)
CHLA-01R-MED % Cell Viability
P
% Cell Viability
Cisplatin
Vincristine
Q

Figure S3. MB tumors express heterogenous ABAT which increases in nutrient scarce microenvironments, Related to Figure 3. **A.** ICC staining of MB cell lines shows UW228-2 and D283 cells exhibit low ABAT expression with no $ABAT_{high}$ cells. Scale Bar, 50µm. **B.** Cerebellar xenograft models recapitulate heterogeneous ABAT expression *in vivo*. Scale bar, 50µm. **C.** ALDH5A1 is homogeneously expressed. Scale bar, 50µm. **D.** ABAT OE cells show significant *SOX9* upregulation, a marker found to indicate metastatic capabilities in MB. t-test. **E.** Single cell RNA sequencing analysis reveals *ABAT* and *ALDH5A1* expression in a subset of circulating tumor cells in the CSF of LMD patients. **F.** mRNA analysis of D425 and CHLA-01R-MED cells in artificial CSF (aCSF) medium shows significant increase in *ABAT* expression. t-test. **G.** ABAT in CHLA-01R-MED cells cultured in either control or aCSF medium shows most cells in aCSF culture have ABAT expression. Scale bar, 50µm. **H.** aCSF cells (n=6) display similar mitochondrial ABAT localization compared to $ABAT_{high}$ cells in control medium. Two-way ANOVA. **I.** Flow cytometry analysis of ABAT reveals MB cells cultured in aCSF have higher overall ABAT expression. **J.** GABA ICC staining shows high co-localization of GABA and mitochondria suggesting MB cells cultured in aCSF utilize GABA as an energy source. Scale bar, 50µm. Magnified image scale bar, 20µm. **K, L.** MB cells cultured in aCSF have significant reduction in Ki67 positive cells (n=6). Scale bar, 50µm. t-test. **M-O.** D283 and D425 Scrambled and CHLA-01R-MED cisplatin and vincristine IC_{50} cytotoxicity values were calculated in order to utilize IC_{50} concentrations for further experiments. **P.** ABAT OE MB cells show significant resistance to standard-of-care chemotherapeutics cisplatin and vincristine (n=3). t-test. **Q.** MB cells cultured in aCSF show significant resistance to standard-of-care chemotherapeutics cisplatin and vincristine (n=3). t-test. n = cells/images quantified (H, L), replicates (P, Q). Histogram data depict mean ± SD. Star Significance: *p<0.05, **p<0.01, ***p<0.001, ****p<0.0001. Two—way ANOVAs were followed by Tukey's multiple comparison test (H).

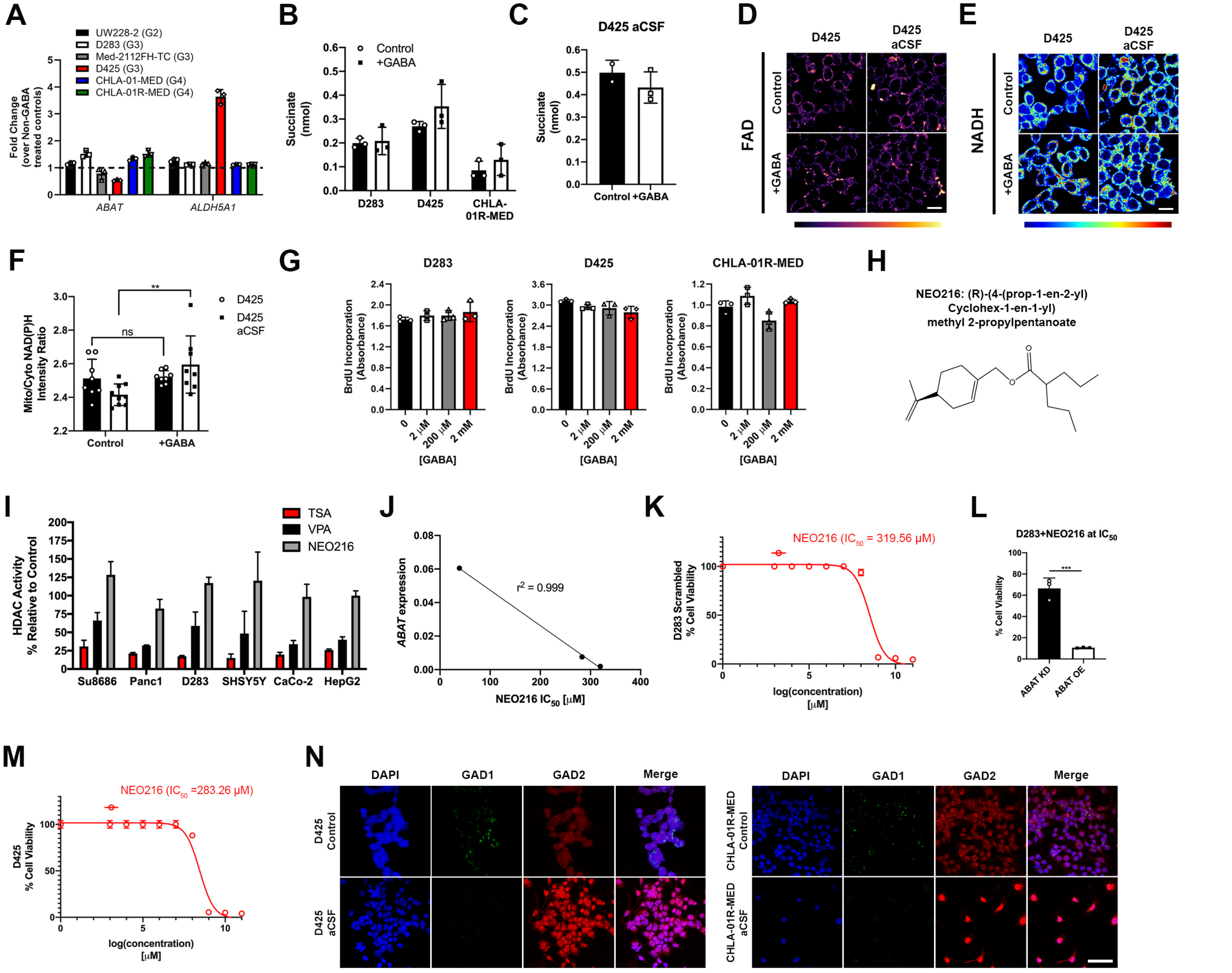

Figure S4. Higher ABAT expression increases GABA metabolism, promotes survival in nutrient-poor conditions, and induces an OXPHOS metabolic phenotype, Related to Figure 5.

A. mRNA analysis of GABA shunt mediators in MB cells after exogenous GABA treatment shows only significant increase in D425 *ALDH5A1* expression. **B.** Quantification of intracellular succinate levels after GABA treatment shows no significant change in cells cultured in control medium (n=3). **C.** Succinate measurement in D425 aCSF cells (n=3) shows no significant change after GABA treatment. **D.** FAD expression is used to map the mitochondria. **E, F.** We find a significant change in mitochondrial NADH, another product of GABA catabolism, in aCSF cells (n=8). Two-way ANOVA followed by Tukey's multiple comparison test. **G.** Exogenous GABA treatment did not affect proliferation (n=3). **H.** Chemical structure of novel ABAT inhibitor NEO216. **I.** Compared to its parent molecule valproic acid (VPA), a known histone deacetylase (HDAC) inhibitor, NEO216 does not show HDAC activity inhibition. **J.** Linear regression line showing NEO216 is more effective in cells with higher *ABAT* expression. **K, L.** Using calculated IC_{50} values for NEO216, we find ABAT OE cells (n=3) are more sensitive to NEO216 than ABAT KD cells (n=3). t-test. **M.** NEO216 IC_{50} value for D425 MB cell line. **N.** Glutamic acid decarboxylase (GAD) isoform 1 and 2 ICC staining reveals increased expression of GAD2 in cells cultured in aCSF medium. Scale bar, 50μm. n = replicates (B, C, G, L), images analyzed (F). Histogram data depict mean ± SD. Star Significance: **$p<0.01$, ***$p<0.001$.

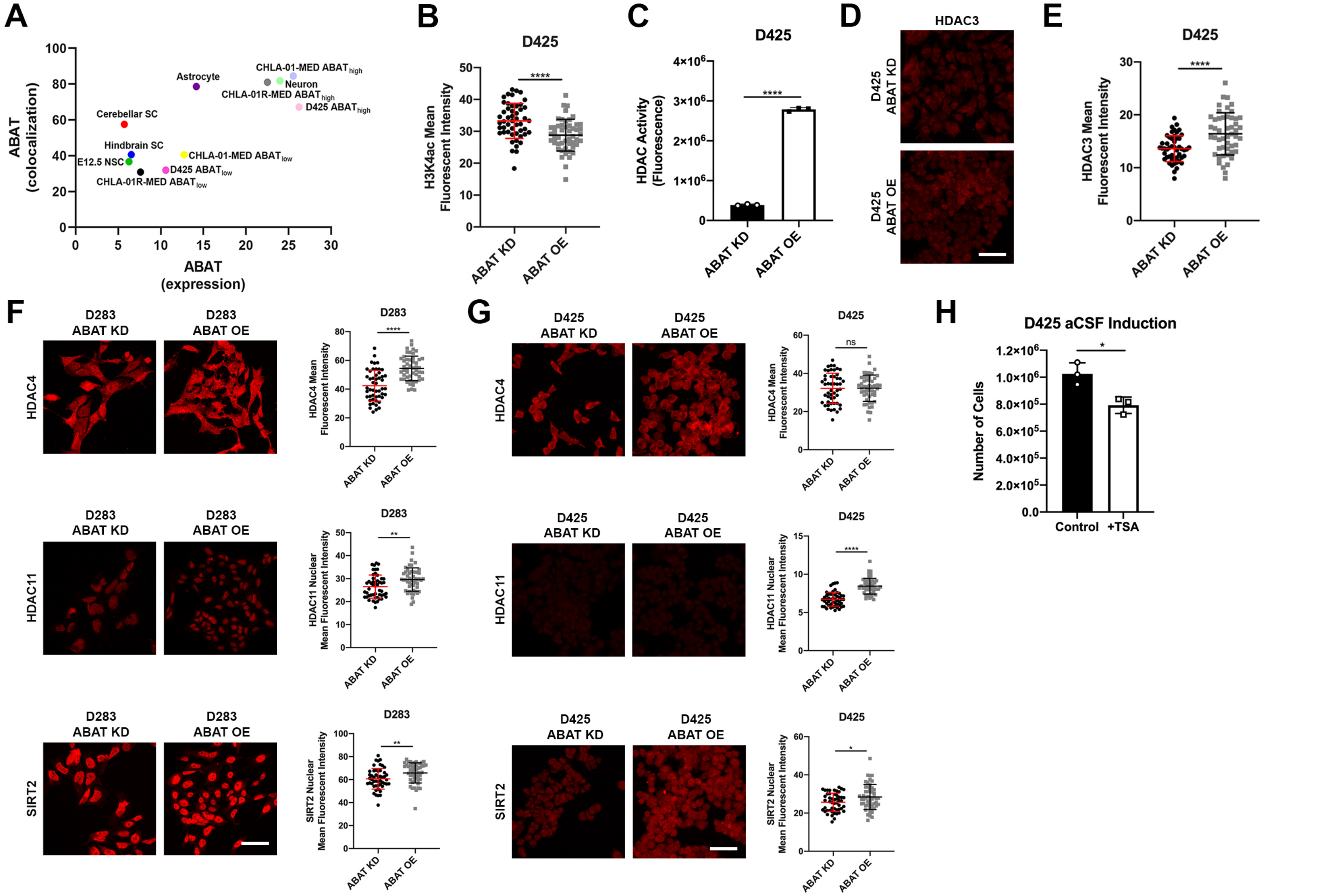

Figure S5. Increased ABAT expression leads to reduced H3K4ac through HDAC3 mediated histone deacetylation, Related to Figure 6. **A.** When screening ABAT expression and localization with mitochondria, ABAT$_{high}$ expressing MB cells cluster with quiescent neurons while ABAT$_{low}$ MB cells clustered with proliferating cells. **B, C.** D425 ABAT OE MB cells (n=50) show significant decrease in H3K4ac expression and a significant increase in HDAC activity compared to ABAT KD cells (n=3). **D, E.** H3K4ac deacetylase HDAC3 shows significant expression increase in D425 ABAT OE cells (n=50). Scale bar, 50μm. **F, G.** HDACs from other classes also show significant upregulation in ABAT OE cells. Scale bar, 50μm. n=50 for all graphs. **H.** HDAC inhibitor TSA treatment on D425 cells being acclimated to aCSF shows significant reduction in viability (n=3) suggesting importance of HDAC in acclimation to reduced nutrient environments. n = nuclei quantified (B, E, F, G), replicates (C, H). Histogram data depict mean ± SD. Star Significance: *$p<0.05$, **$p<0.01$, ****$p<0.0001$, t-test.
