# Supplementary material for: Medulloblastoma uses GABA transaminase to survive in the cerebrospinal fluid microenvironment and promote leptomeningeal dissemination
Source: Cell Rep. Author manuscript; Available in PMC 2022 Jun 29. (PMC8848833; doi:10.1016/j.celrep.2021.109302)
Supplement: Supplementary table [file NIHMS1774599-supplement-Supplementary_table.docx]

Table S1. Primer List

| *Abat* (NM_001170978), Exon Location 2-3 | Integrated DNA Technologies | Assay Name: Mm.PT.58.8192334 |
| --- | --- | --- |
| *ABAT* (NM_000663), Exon Location 15-16 | Integrated DNA Technologies | Assay Name: Hs.PT.58.3572402 |
| *Aldh5a1* (NM_172532), Exon Location 4-5 | Integrated DNA Technologies | Assay Name: Mm.pT.58.8466382 |
| *ALDH5A1* (NM_170740), Exon Location 6-7 | Integrated DNA Technologies | Assay Name: Hs.PT.58.39901716 |
| *CCNB2* (NM_004701), Exon Location 5-6 | Integrated DNA Technologies | Assay Name: Hs.PT.58.39618525 |
| *CCNF* (NM_001761), Exon Location 5-7 | Integrated DNA Technologies | Assay Name: Hs.PT.56a.3391893 |
| *CDC25A* (NM_201567), Exon Location 1-3 | Integrated DNA Technologies | Assay Name: Hs.PT.58.800341 |
| *CDC25C* (NM_001790), Exon Location 2-7 | Integrated DNA Technologies | Assay Name: Hs.PT.58.15047788 |
| *CDK1* (NM_001170407), Exon Location 3-4 | Integrated DNA Technologies | Assay Name: Hs.PT.58.40377349.g |
| *CDKN1A* (NM_000389), Exon Location 4-5 | Integrated DNA Technologies | Assay Name: Hs.PT.58.40874346.g |
| *CDKN2A* (NM_001195132), Exon Location 5-5 | Integrated DNA Technologies | Assay Name: Hs.PT.58.40743463.g |
| *SOX9* (NM_000346), Exon Location | Integrated DNA Technologies | Assay Name: Hs.PT.58.38984663 |
| *Actb* (NM_007393), Exon Location 4-5 | Integrated DNA Technologies | Assay Name: Mm.PT.58.33257376.gs |
| *ACTB* (NM_001101), Exon Location 1-2 | Integrated DNA Technologies | Assay Name: Hs.PT.39a.22214847 |
